# Sex Differences in Carbohydrate Metabolism Are Linked to Gene Expression in *Caenorhabditis elegans*

**DOI:** 10.1371/journal.pone.0044748

**Published:** 2012-09-11

**Authors:** Claudia Miersch, Frank Döring

**Affiliations:** Department of Molecular Prevention, Institute of Human Nutrition and Food Science, Christian-Albrechts-University of Kiel, Kiel, Germany; Wageningen University, The Netherlands

## Abstract

The male and the hermaphrodite forms of the nematode *Caenorhabditis elegans* (*C. elegans*) differ markedly in anatomy, nervous system and behavior at adulthood. Using the male mutants *fog-2*, *him-5*, and *him-8,* we compared body proportions and composition, and aspects of carbohydrate metabolism and gene expression between the *C. elegans* sexes in three adult stages. In all experiments, both sexes were grown on the same plate and separated using flow cytometry. The fat to fat-free mass ratio and the body volume-adjusted fat mass is similar between the sexes, although the body size is more than 50% smaller in adult males than in age-matched hermaphrodites. The volume-adjusted total RNA content is approximately 2-fold lower in males. Biochemical and NMR-based analyses reveal higher trehalose levels and much lower glucose levels in males than in hermaphrodites. The resulting trehalose-to-glucose ratio is 5.4-fold higher in males. These sex differences are reflected in gene expression data because the genes encoding key enzymes of the glycolysis and trehalose synthesis pathways are more highly expressed in males than in hermaphrodites. Notably, expression of the phosphofructokinase gene (C50F4.2) is 29-fold higher in males. Comparative analysis of gene expression data identifies 285 male-specific and 160 hermaphrodite-specific genes. These include transcription factor and C-type lectin-encoding genes. More than 35% of all C-type lectin genes are more highly expressed in males. The expression of many C-type lectin genes differs by a factor of >100 between the sexes. In conclusion, we found sex differences in carbohydrate metabolism that are linked to gene expression and identified certain lectin genes that are differentially expressed by the *C. elegans* sexes.

## Introduction

The nematode *Caenorhabditis elegans* (*C. elegans*) consists of two sexes: a self-fertilizing hermaphrodite (diploid X chromosome) and a conventional male (single X chromosome). Hermaphrodites are the dominant sex; however, males are generated at a low frequency (approximately 1 in 500, <0.3%) by spontaneous X chromosome non-disjunction events occurring during meiosis [1]. Mating produces a uniform number of males and hermaphrodites because the larger male sperm replace hermaphrodite sperm during fertilization, which results in an equal segregation of sex chromosomes [2], [3]. Although males are smaller than hermaphrodites, they contain more somatic cells (1031 in males and 959 in hermaphrodites) [4], [5]. Most physical differences between the sexes occur during larval development because males and hermaphrodites are nearly identical upon hatching. Beginning at the L4 larval stage, the two sexes are distinguishable using stereomicroscopy. At this stage, the blunt-ended tail of the male starts to develop. The postembryonic development of sex-specific cell lineages and differentiated cells has been previously examined [4], [6]. Furthermore, the anatomy of the male differs from that of the hermaphrodite by having one gonadal arm and having specialized muscles and neurons [7]. Adult hermaphrodites have a peaked tail, a vulva, a uterus and two gonadal arms, which produce sperm and oocytes. The pharynx, excretory system and main body muscles do not exhibit sexually dimorphic characteristics [4], [5], [8].

Due to the existence of sex-specific neurons and sex-related differences in the core nervous system, *C. elegans* exhibit a sexually dimorphic nervous system [9]. As a result, males and hermaphrodites possess sex-specific behaviors, such as hermaphrodite egg-laying behavior and male mating behavior [10]–[12]. Furthermore, food-leaving behavior, locomotion, olfaction, learning and memory capabilities are different between the *C. elegans* sexes [13]–[16]. Food-leaving behavior occurs in males when they are cultivated on plates with an adequate food source in the absence of hermaphrodites; in contrast, hermaphrodites remain on plates when no males are present [14]. Lipton and coworkers [14] concluded that males attempt to find sexual partners for mating, whereas hermaphrodites do not need to mate for reproduction.

Physiological sex differences also exist, such as lifespan, stress response and immune resistance. In many mammals, including humans, females live longer than males [17]. In *C. elegans*, the lifespan of both sexes strongly depends on the culture conditions and social interactions. Cultivating males and hermaphrodites on agar plates at 20°C results in median lifespans of 10 and 17 days, respectively [18]. However, when males are cultivated individually, the male median lifespan becomes longer than that of the hermaphrodite by 20%. This increase in lifespan is possibly due to the lack of male-male interaction and other, unknown aspects related to locomotion. Additionally, mating dramatically reduces the median lifespan of hermaphrodites from 17 to 8 days [19]. In terms of oxidative stress, males are much more sensitive to hydrogen peroxide and paraquat than hermaphrodites due to the sex-specific transcription factor *mab-3* [20]. In contrast, males are more resistant to the fungal pathogen *Cryptococcus neoformans* than hermaphrodites, and this resistance is dependent on the *daf-16* transcription factor [21]. Sex differences are also visible at the molecular level, as indicated by gene expression analyses [22]–[24], which focused on gene expression and sex differences during larval development and cell differentiation.

The average body size of an adult male is approximately threefold smaller than that of an adult hermaphrodite [25]. Chemically, nematodes consist primarily of water (almost 85%) [26], followed by proteins, lipids, carbohydrates, nucleic acids and minerals. Adult hermaphrodites have a triglyceride content that is five- to tenfold lower than the protein content [27]–[29]. The carbohydrate trehalose is present in higher concentrations than glucose, since it is thought to function in the transport of soluble sugar [30]–[33]. Little information is available regarding the body composition and carbohydrate metabolism of *C. elegans* males. Thus, we systematically compared parameters relating to body proportion and composition, trehalose content, glucose content, total RNA content and gene expression data in males and hermaphrodites. As a final goal of our study, we attempted to link physiological sex differences to gene expression in *C. elegans.*

## Results

### N2, *fog-2*, *him-8*, *him-8 GFP* and *him-5* Males are Similar in Morphology

**Figure 1:**
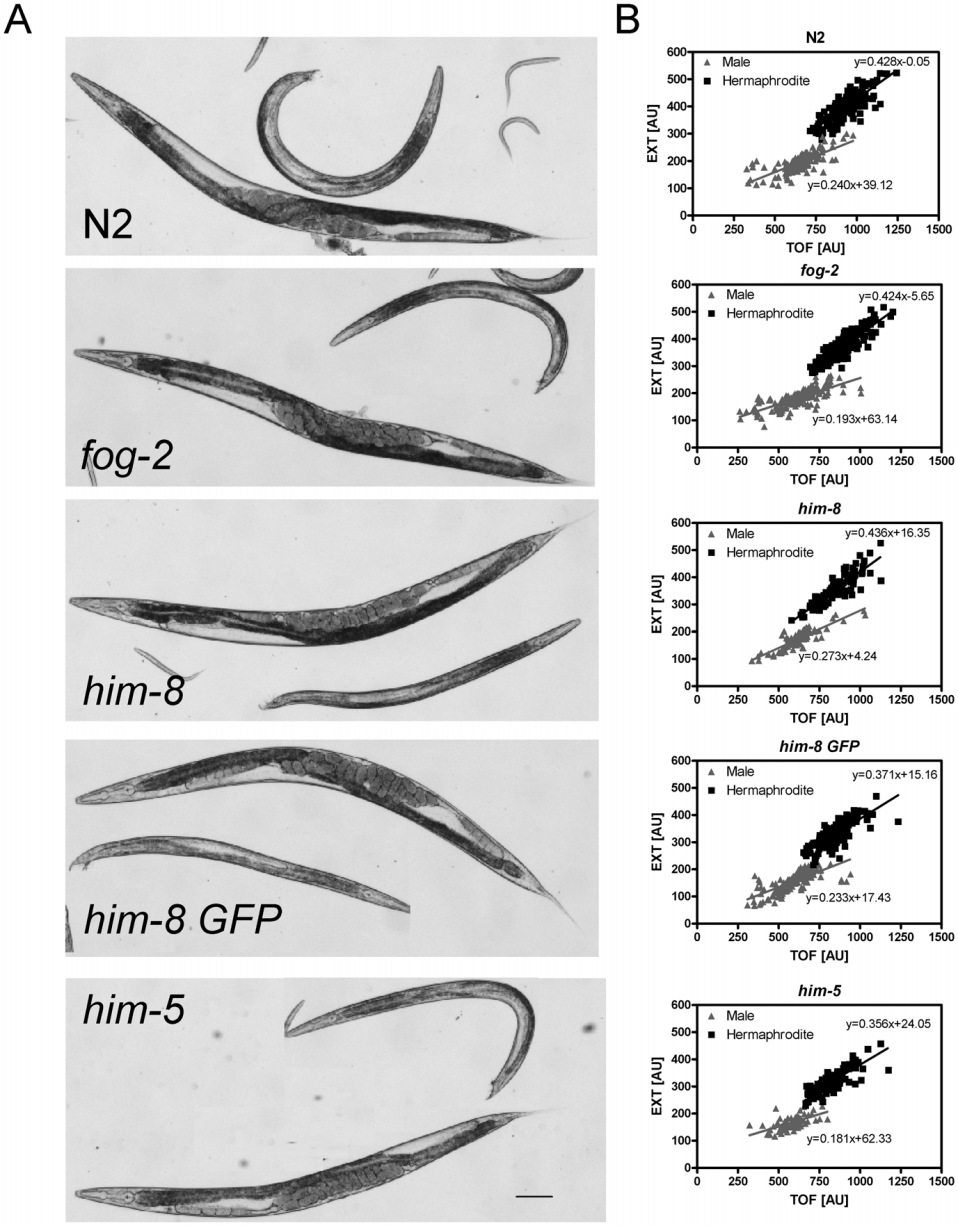
The morphology (A) and flow cytometry-based separation (B) of adult males and hermaphrodites in various *C. elegans* male mutants. The mutants were synchronized by hypochlorite treatment of gravid worms and analyzed at one day of adulthood (90 h). (A) Microscopic pictures of wild type (N2) and male mutants. Two independent experiments were performed with 12–24 worms per sex and strain. Scale bar: 100 µm. (B) Flow cytometry displays the time of flight (TOF) and extinction (EXT) values for males and hermaphrodites in the N2 strain and in various mutants. Representative results from two independent experiments with at least 160 worms per strain are shown. Each point represents a single worm. Linear regression lines show differences in TOF and EXT between males and hermaphrodites. The indicated equations correspond to the regression lines.

To examine sex differences in *C. elegans*, *him* (*h*igh *i*ncidence of *m*ales) and *fog* (*f*eminization *o*f *g*ermline) mutants were used; these mutants produce higher proportions of males in comparison to N2 wild type hermaphrodites. N2 males, which were obtained in higher frequency by mating, were also utilized in initial control experiments. Mutations in the *him-8* and *him-5* genes result in a higher incidence of males due to meiotic X chromosome loss. Hermaphrodites of *him-8 (e1489)* IV and *him-5 (e1490)* V mutants produce more than 30% males [1], [34]. The transgenic mutants *him-8 (e1489)* IV; *nls128* II (abbreviated: *him-8 GFP*) express GFP under the control of the *pkd-2* promoter in male-specific neurons [35]. *fog-2* hermaphrodites are functionally classed as females because they do not produce sperm [36]; therefore, in this strain, a high proportion of males is maintained through mating. To compare the morphology of selected male *fog-2*, *him-8*, *him-8 GFP* and *him-5* mutants with N2 wild type individuals, adult worms were examined using stereomicroscopy. We found no obvious differences in behavior between the mutants and the N2 nematodes. In all mutants analyzed, both sexes were similar in general anatomy and morphology when compared to N2 wild type individuals (**Figure 1A**). Thus, the male mutants were suitable for studying certain physiological sex differences in *C. elegans*.

### Flow Cytometry-based TOF and EXT Values are Proxies for Body Length and Body Volume

**Figure 2:**
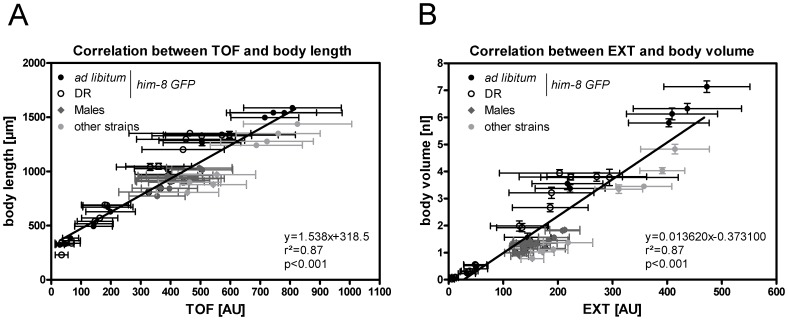
The relationships between body length and time of flight (TOF, A) and between body volume and extinction (EXT, B). The data include the different feeding conditions (*ad libitum* or dietary restriction), both sexes, different strains (N2, *fog-2*, *him-8*, *him-8 GFP* and *him-5*) and different developmental stages. Each data point represents the mean ± SDs from one experiment. In each experiment, at least 10 and 140 worms were analyzed via microscopy and flow cytometry, respectively. The linear regression equations, p and r^2^ values were calculated.

In this study, the flow cytometry-based parameters of time of flight (TOF) and extinction (EXT) were used for the basal characterization of males and hermaphrodites. To confirm that the TOF and EXT values reflect the axial length and body volume of the worms [27], respectively, these flow cytometry-based parameters were compared with body size data obtained from microscopic images. To cover a wide range of body proportion values, different male mutants, different developmental stages and different feeding conditions (*ad libitum* or dietary restriction) were included in the analysis. Significant relationships between TOF and body length (r^2^ = 0.87, p<0.001, **Figure 2A**) and between EXT and body volume (r^2^ = 0.87, p<0.001, **Figure 2** **B**) were identified. Moreover, based on the obtained TOF and EXT values, adult males and hermaphrodites from different male mutants were clearly distinguishable from one another and clustered in well-defined clouds (**Figure 1** **B**). Therefore, flow cytometry-based TOF and EXT values are good proxies for body length and body volume. In addition, our flow cytometry approach would allow the separation of males and hermaphrodites to unravel sex differences in body proportions in large *C. elegans* populations.

### The body Size of Males is Similar to that of Hermaphrodites during Larval Stages but Smaller at Adulthood

**Figure 3:**
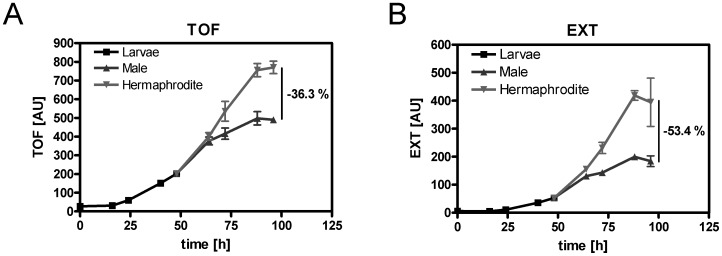
The flow cytometry-based parameters measuring the body proportions of *him-8 GFP* males and hermaphrodites at various developmental stages. The time of flight (TOF, A) and extinction (EXT, B) values of *him-8 GFP* worms were assessed via flow cytometry at various developmental stages from egg [0 h] to adult [96 h]. The *him-8 GFP* males and hermaphrodites could be separated once they reached the young adult stage (approximately 64 h) based on the GFP signal. The relative differences between males and hermaphrodites are given at 96 h. The results from three independent experiments are presented as the mean ± SDs. At least 140 worms per sex and time point were used in each experiment.

**Figure 4:**
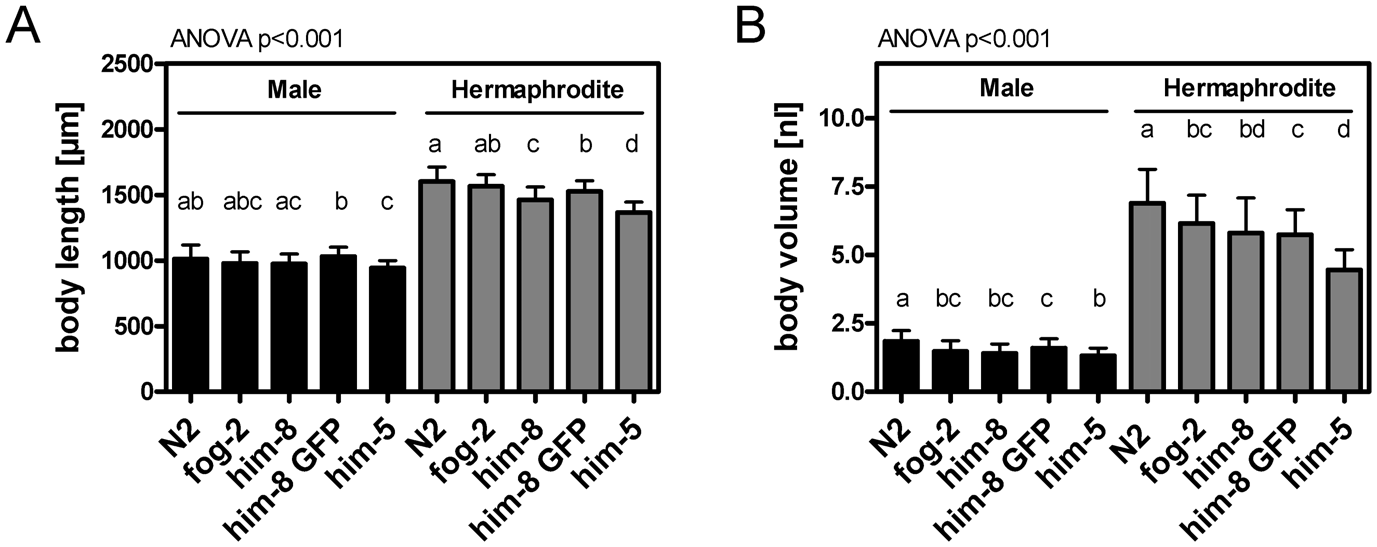
The body length (A) and body volume (B) of adult males and hermaphrodites in various *C. elegans* male mutants. The mutants were synchronized by hypochlorite treatment of gravid worms and analyzed at one day of adulthood (90 h). Microscopic images from each mutant strain were used to determine the body length and body volume of males and hermaphrodites. The values are expressed as the mean ± SDs and include two independent experiments. Between 12–24 worms per sex and strain were scored in each experiment. The bars with different letters represent significantly different results between strains (p<0.05). To determine significant differences, a one-way ANOVA was performed and followed by a post hoc test (the Bonferroni multiple comparison test or Dunnett’s T3 test, when inhomogeneity of variance was evident).

As mentioned earlier, males are almost indistinguishable from hermaphrodites upon hatching. During development, morphological differences between the sexes become increasingly apparent [7]. Thus, the body length (TOF) and body volume (EXT) of *him-8 GFP* males were compared with *him-8 GFP* hermaphrodites at the larval and adult stages (**Figures 3A and B**). At the young adult stage (64 h), males and hermaphrodites exhibited almost identical body sizes. As expected, sex differences in body size became increasingly evident as the worms reached adulthood. At day one of adulthood (96 h), *him-8 GFP* males had a 36.3% shorter body length (TOF) and a 53.4% smaller body volume (EXT) than age-matched hermaphrodites. These results were confirmed by microscopy data (**Figures 4** **A and B**). Therefore, substantial differences in body proportions exist between the sexes at adulthood but not during larval stages.

### The Fat to Fat-free Mass Ratio and the Volume-adjusted Fat Mass of Adult Males are Similar to those of Adult Hermaphrodites

**Figure 5:**
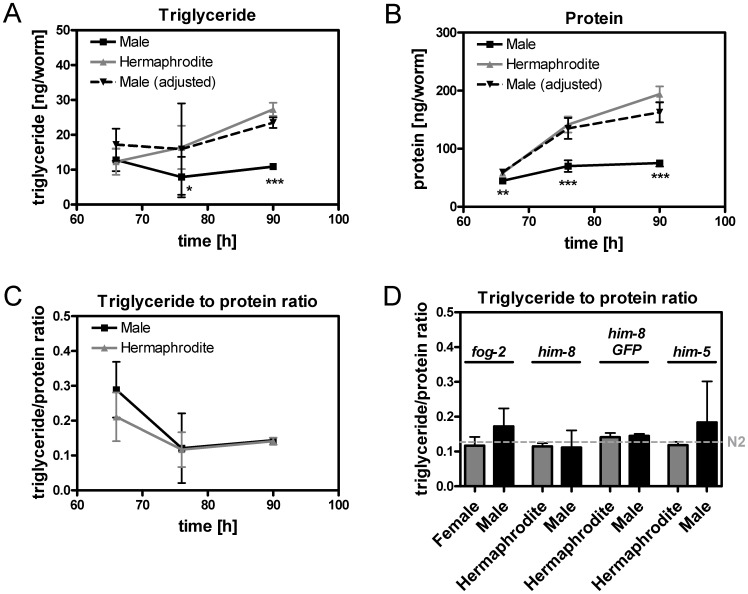
The body composition of males and hermaphrodites at various developmental stages. The triglyceride content (A), protein content (B) and the resulting triglyceride-to-protein ratio (C) of *him-8 GFP* males and hermaphrodites at young adulthood (66 h), adulthood (76 h) and one day of adulthood (90 h). Male body composition parameters were normalized to the body volume so that they were comparable to the results for the hermaphrodites (A, B). Comparison of the triglyceride-to-protein ratio for the *fog-2*, *him-8*, *him-8 GFP* and *him-5* mutants at adulthood (90 h, D). The results of N2 hermaphrodites were used as a reference and included as a dashed line (D). Data represent the mean ± SDs from 3–8 independent experiments. Significant differences between males and hermaphrodites are indicated using asterisks (* p<0.05, ** p<0.01, *** p<0.001, *t*-test).

As body proportions of adult worms show sex differences, body composition may also differ. To test this hypothesis, the triglyceride and protein content, which represents the fat and fat-free mass of *C. elegans* [27], respectively, was determined in *him-8 GFP* males and hermaphrodites at young adulthood (66 h), adulthood (76 h) and one day of adulthood (90 h). As shown in **Figures** **5A and B**, males exhibited significantly lower triglyceride and protein content per worm at all three developmental stages than did hermaphrodites (see also **Table S1**).

Physiologically, the proportions of fat and protein in relationship to body volume and fat to fat-free mass ratio are of central importance when evaluating the body composition of an organism [27]. As shown in **Figures 5** **A and B**, normalizing the male triglyceride and protein values to body volume no apparent differences were visible between *him-8 GFP* mutants of both sexes. This result was confirmed by the observation that the triglyceride-to-protein ratio was not significantly different between *him-8 GFP* males and hermaphrodites at all three adult stages (**Figure 5** **C**). Additionally, the triglyceride-to-protein ratios between males and hermaphrodites of *fog-2*, *him-8* and *him-5* mutants were not different at adulthood (90 h, **Figure 5** **D, Table S1**). The ratios observed for hermaphrodites are similar to those reported in the literature [27]–. In summary, the body compositions of male and hermaphrodite *C. elegans* individuals are similar at all three adult stages.

### The Trehalose-to-glucose Ratio is Higher in Adult Males than in Adult Hermaphrodites

**Figure 6:**
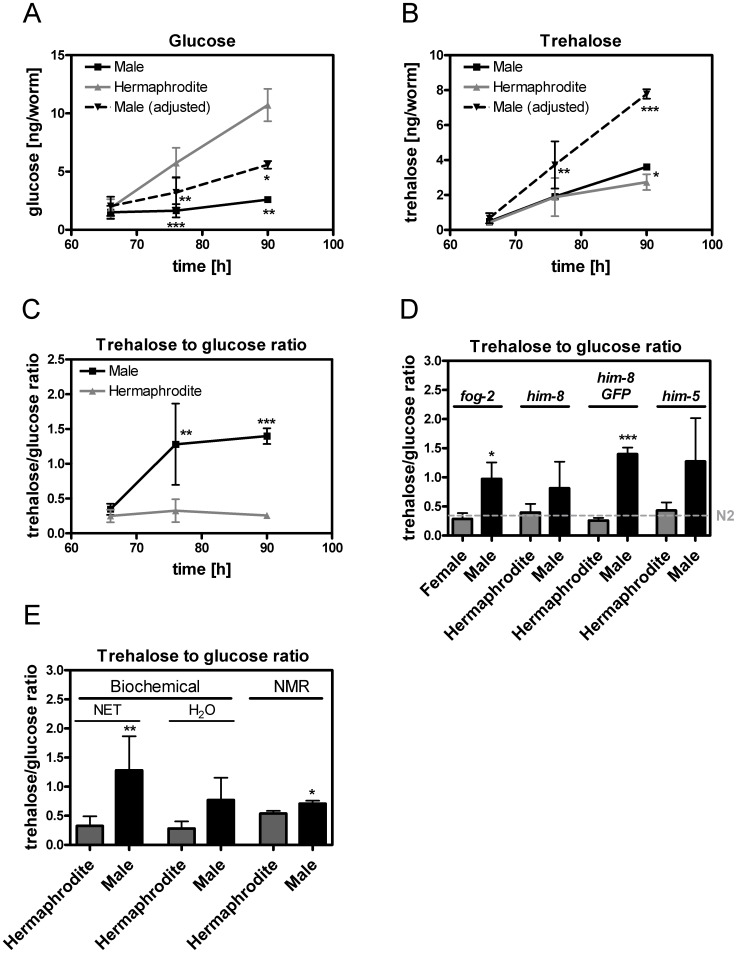
The trehalose and glucose content of males and hermaphrodites at various developmental stages. The glucose content (A), trehalose content (B) and resulting trehalose-to-glucose ratio (C) of *him-8 GFP* males and hermaphrodites at young adulthood (66 h), adulthood (76 h) and one day of adulthood (90 h) are shown. The male glucose and trehalose content was adjusted to the body volume so that it was comparable to the results for the hermaphrodites (A, B). Comparison of the trehalose-to-glucose ratio in the *fog-2*, *him-8*, *him-8 GFP* and *him-5* mutants at adulthood (90 h, D). The results of N2 hermaphrodites were used as a reference and included as a dashed line (D). The trehalose and glucose levels were validated using NMR spectroscopy (E). The trehalose-to-glucose ratio from biochemical assays (using NET buffer or distilled water as solvent) and NMR spectroscopy experiments are presented. The data represent the mean ± SDs from 3–8 independent experiments. Significant differences between males and hermaphrodites are indicated using asterisks (* p<0.05, ** p<0.01, *** p<0.001, *t*-test).

To gain insight into metabolic differences between the sexes, the glucose and trehalose content of *him-8 GFP* males and hermaphrodites was analyzed at young adulthood (66 h), adulthood (76 h) and one day of adulthood (90 h). As shown in **Figure 6** **A**, adult males had significantly lower glucose levels than hermaphrodites, even after adjusting the male glucose values for body volume (see **Table S2**). The adult *him-8 GFP* males exhibited equal (66 h, 76 h) or higher (90 h) trehalose levels than did the adult hermaphrodites (**Figure 6** **B**). The trehalose content obtained from young adult hermaphrodites is somewhat lower than reported values [37], [38]. After adjustment of the trehalose content to the body volumes, *him-8 GFP* males contained significantly higher trehalose levels than hermaphrodites at all adult stages (**Figure 6** **B**). The contrasting glucose and trehalose levels in the males and hermaphrodites resulted in 1.4-(66 h), 3.9-(76 h) and 5.4-fold (90 h) higher trehalose-to-glucose ratios in adult males compared to those in hermaphrodites (**Figure 6** **C**). This difference between the sexes was also present in *fog-2, him-8* and *him-5* mutants (**Figure 6** **D, Table S2**). Consistent with the biochemical data, a significantly higher trehalose-to-glucose ratio was found in males compared to hermaphrodites using NMR spectroscopy (**Figure 6** **E**).

### The Total RNA Content is Lower in Adult Males than in Adult Hermaphrodites

**Figure 7:**
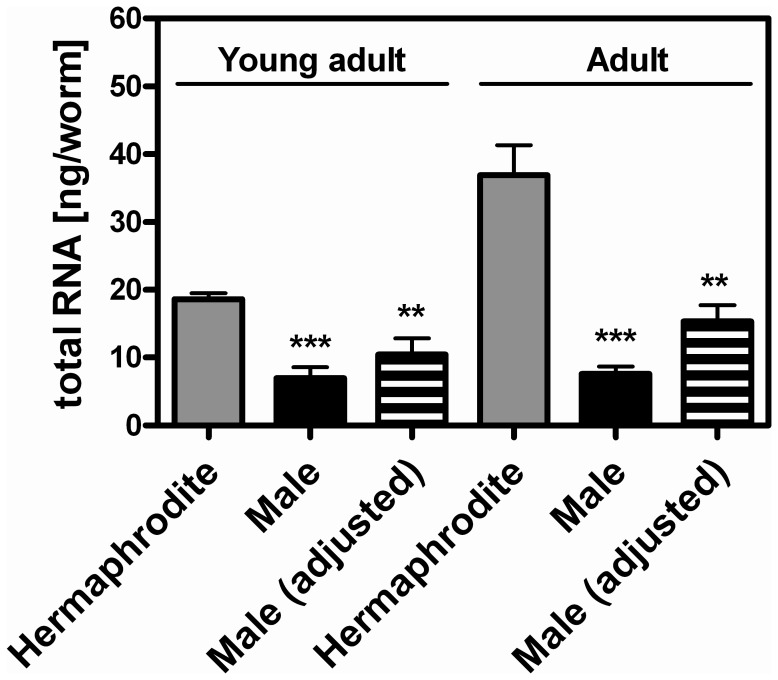
The total RNA content of young adult and adult *him-8 GFP* males and hermaphrodites. Total RNA was isolated from young adult (66 h) and adult (76 h) *him-8 GFP* males and hermaphrodites. The RNA content of males was adjusted to body volume so that the results were comparable to those of hermaphrodites. The data represent the mean ± SDs, n = 3. Significant differences between the male or male (adjusted) and the hermaphrodite were determined using a *t*-test (**p<0.01, ***p<0.001).

As part of our gene expression analysis, the total RNA content of *him-8 GFP* males and hermaphrodites was determined when these worms reached young adulthood (66 h) and adulthood (76 h). Interestingly, a 2.7- to 4.8-fold lower total RNA content per worm was found in the males than in the hermaphrodites (**Figure 7**). This difference was also evident after adjusting the total RNA content of the males for body volume (1.8- to 2.4-fold lower). Additionally, the total RNA content of the hermaphrodites increased significantly from the young adult to the adult stage, but did not change in males during this period.

### Identification of Male- and Hermaphrodite-specific Genes

**Figure 8:**
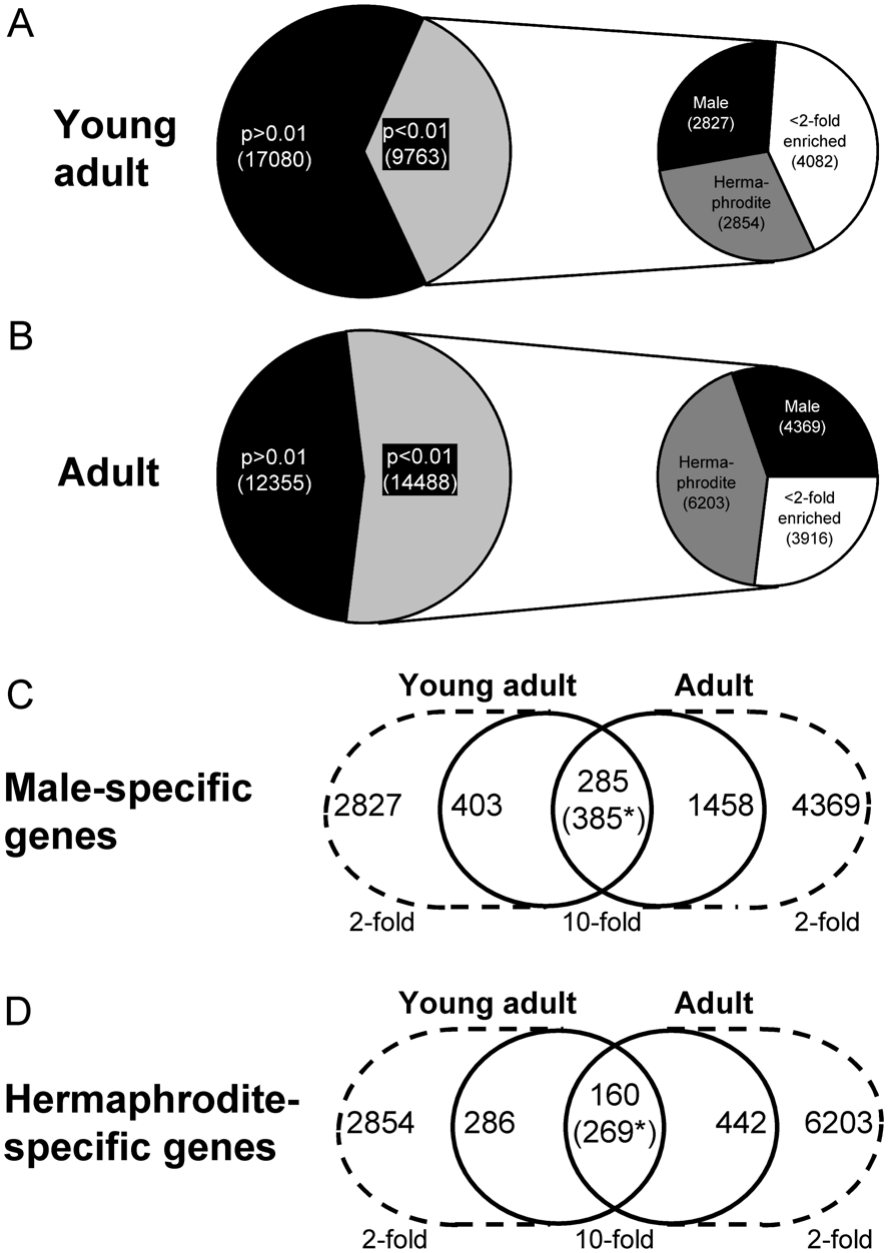
The number of differentially expressed genes between the *him-8 GFP* males and hermaphrodites. The pie charts (A, B) show the number of differentially expressed genes between the *him-8 GFP* males and hermaphrodites at the young adult (66 h, A) and adult (76 h, B) stages. Genes that were expressed 2-fold or more higher in the corresponding sex were considered as male-enriched (black) or hermaphrodite-enriched (grey) genes. The number of genes is listed within the brackets. The Venn diagrams (C, D) display the overlap of male- (C) or hermaphrodite- (C) specific genes at the young adult (66 h) and adult (76 h) stages. To scale down the number of genes, the cut-off was increased from 2-fold (dashed cycles) to 10-fold (solid circles). * Genes in the other sex that were expressed at levels that was higher than 100 were excluded.

To identify genes that were differentially expressed in both sexes, comparative microarray analysis was performed for *him-8 GFP* males and hermaphrodites in young adult (66 h) and adult (76 h) worms. After analyzing 26843 genes, 9763 genes were expressed at significantly different (p<0.01) levels between both sexes at the young adult stage (adult stage: 14488 genes, **Figures 8** **A and B**). Of these genes, 2827 (adult: 4369 genes) were expressed at 2-fold higher levels or greater in males than in hermaphrodites (male-enriched genes). Additionally, 2854 genes (adult: 6203 genes) were considered as hermaphrodite-enriched. Next, we searched for genes that were at least 10-fold more highly expressed in one sex at both adult stages and that exhibited an expression level of <100 in the other sex. These genes were considered sex-specific and included 285 male-specific and 160 hermaphrodite-specific genes (**Figures 8** **C and D**). Many of these genes were not annotated; only male- and hermaphrodite-specific genes that could be assigned to a gene class were included in **Tables 1** **and** **2**. The full data showing all of the genes examined are given in **Tables S3** (male-specific) **and S4** (hermaphrodite-specific).

**Table 1:** Male-specific genes^a^.

Gene class	Gene name	Molecular function^b^	Male to hermaphroditeratio young adult (adult)	P-value young adult (adult)
C-type lectin	*clec-92 clec-93* *clec-95* *clec-96* *clec-102* *clec-107* *clec-108* *clec-109* *clec-110* *clec-113* *clec-116* *clec-125* *clec-126* *clec-129* *clec-130* *clec-132* *clec-133* *clec-134* *clec-135* *clec-136* *clec-141* *clec-157* *clec-158* *clec-159* *clec-161* *clec-164* *clec-176* *clec-179* *clec-181* *clec-183* *clec-193* *clec-213* *clec-217* *clec-219* *clec-232* *clec-250* *clec-254* *clec-256* *clec-257* *clec-259* *clec-261* *clec-263*	protein interactions (some genesfrom this class are defense-related)	279.5 (935.9)43.0 (109.7)220.8 (925.5)257.5 (898.3)20.6 (62.8)903.5 (1582.3)145.8 (508.9)132.6 (1046.5)186.2 (816.1)13.9 (12.0)28.0 (33.3)176.6 (404.7)121.8 (171.5)496.1 (1421.1)341.0 (772.0)140.9 (167.2)220.9 (1046.3)1593.0 (1063.3)582.3 (1292.6)139.4 (1309.1)185.7 (541.8)356.9 (201.2)189.4 (925.3)145.5 (198.7)267.2 (952.3)24.4 (35.4)12.2 (10.8)23.0 (52.9)256.1 (729.2)247.9 (578.6)57.3 (65.2)34.1 (44.6)316.0 (1447.9)183.1 (1163.3)252 2 (633.3)19.8 (15.4)42.9 (21.1)19.3 (33.8)33.8 (51.9)148.9 (238.4)232.0 (1092.4)320.2 (999.5)	<0.001 (0.009)0.002 (<0.001)0.001 (0.005)0.002 (<0.001)<0.001 (<0.001)0.003 (<0.001)0.002 (0.001)0.002(<0.001)0.005 (0.006)<0.001 (<0.001)0.003 (0.004)0.003 (0.002)0.004 (<0.001)<0.001 (<0.001)0.003 (<0.001)<0.001 (0.004)0.006 (<0.001)0.001 (0.002)0.001 (<0.001)<0.001 (0.002)0.001 (<0.001)<0.001 (<0.001)<0.001 (<0.001)0.002 (0.006)0.002 (<0.001)0.002 (<0.001)0.004 (0.004)0.006 (0.008)<0.001 (0.001)<0.001 (<0.001)<0.001 (0.002)0.003 (<0.001)0.001 (<0.001)<0.001 (0.002)0.002 (0.002)0.003 (<0.001)0.004 (0.002)0.002 (0.007)0.008 (0.001)0.001 (0.006)0.004 (0.006)0.002 (0.003)
galactose-binding lectin	*F46A8.3* *F46A8.5* *F46A8.8* *F49F1.10* *F49F1.11* *F49F1.9*	sugar binding	312.0 (855.3)337.1 (1335.6)366.4 (1046.8)251.4 (1195.6)323.8 (1330.1)460.5 (884.5)	0.007 (0.002)<0.001 (<0.001)<0.001 (<0.001)0.009 (<0.001)0.004 (0.003)<0.001 (0.001)
fungus-induced protein (fungus induced protein related)	*fipr-16*	antimicrobial activity	307.8 (792.9)	0.005 (<0.001)
SCP-like extracellular protein	*scl-8* *scl-18*	defense-related protein containingSCP domain	538.6 (1034.2)159.7 (1323.1)	0.001 (0.004)0.006 (<0.001)
hypersensitive to pore-forming toxin	*hpo-33* *hpo-37*	immune defense	159.8 (1352.6)462.4 (606.1)	0.002 (<0.001)0.004 (0.008)
polycystins and coexpressed genes	*cwp-2* *cwp-3* *lov-1* *pkd-2*	neuropeptide signaling/transmembranetransport	54.5 (339.3)44.6 (110.4)55.6 (111.6)41.1 (120.3)	0.001 (<0.001)0.002 (0.003)0.004 (0.003)0.001 (0.002)
Nematode astacin protease	*nas-17* *nas-19*	astacin-like metalloprotease	598.6 (1335.3)337.8 (1556.8)	0.002 (<0.001)0.001 (<0.001)
lipase related	*lips-2*	triacylglycerol lipase	203.9 (638.2)	0.006 (0.002)
insulin related	*ins-25*	hormone activity	17.8 (18.7)	0.002 (0.002)
collagen and related protein	*col-183*	structural constituent of cuticle	77.0 (264.2)	0.001(<0.001)
ribosomal protein	*rpl-11.2*	structural constituent of ribosome	144.4 (1826.8)	<0.001 (<0.001)
SET (trithrax/polycomb) domain containing protein	*set-15*	histone H3 methyltransferase	80.0 (201.9)	0.002 (0.002)
trypsin like protease	*try-5*	endopeptidase activity	41.5 (76.5)	<0.001 (0.004)
transpliced leader sequence	*sls-2.18*	unknown	16.1 (22.9)	<0.001 (<0.001)
Innexin	*inx-1*	unknown	18.4 (21.1)	0.004 (0.006)
serpentine receptor, class D, class G, class I, class H, class V, class Z	*srg-1* *sri-25* *srh-287* *srd-1* *srz-93* *srv-11*	receptor like protein olfactory receptor chemosensory receptor pseudogene transmembrane receptor activity	14.4 (16.2)10.5 (41.3)13.1 (13.0)15.7 (14.0)13.4 (16.1)10.9 (17.4)	0.008 (0.008)<0.001 (0.005)<0.001 (0.003)0.004 (0.008)0.001 (0.002)0.004 (0.008)
seven TM receptor	*str-55*	olfactory receptor	87.2 (46.2)	0.005 (0.001)
male abnormal	*mab-23* *mab-3*	transcription factor activity	22.7 (43.5)25.0 (44.0)	<0.001 (<0.001)<0.001 (0.003)
C. elegans homeobox	*ceh-7*	transcription factor activity	10.9 (11.2)	0.003 (<0.001)
dystroglycan	*dgn-3*	unknown	39.6 (55.7)	<0.001 (<0.001)
MX region of TRA-2 related	*xtr-2*	protein-protein interaction, regulating negatively tra-2	14.3 (26.4)	0.002 (<0.001)
FMRF-like peptide	*flp-23*		109.4 (959.2)	0.002 (<0.001)
TNF receptor associated factor (TRAF) homolog	*trf-1*	signal transduction, regulation of apoptosis	28.1 (40.9)	0.003 (0.004)
nuclear hormone receptor family	*nhr-266* *nhr-113*	transcription factor activity	16.4 (13.9)16.6 (72.5)	0.009 (0.008)<0.001 (0.002)
cytochrome P450 family	*cyp-35B1*	oxidoreductase, electron carrier activity, monooxygenase activity	28.0 (12.9)	0.007 (<0.001)
neuropeptide-like protein	*nlp-22*	unknown	32.4 (60.4)	<0.001 (0.006)
F-box A protein	*fbxa-204*	pseudogene	63.0 (91.0)	<0.001 (<0.001)

a supplemental data contain a complete list of all genes (also unknown genes and genes without a gene class).

b sometimes the function of a gene is not precisely defined or only described as “predicted” function, information about genes was obtained from WormBase (WS229, www.wormbase.org).

**Table 2:** Hermaphrodite-specific genes^a^.

Gene class	Gene name	Molecular function^b^	Hermaphrodite to maleratio young adult (adult)	P-valueyoung adult (adult)
F-box B protein	*fbxb-67*	protein-protein interaction(ubiquitin protein degradation pathway)	12.9 (37.3)	<0.001 (<0.001)
F-box C protein	*fbxc-31* *fbxc-32* *fbxc-42*		12.8 (57.4)84.1 (191.9)29.7 (151.9)	0.005 (0.002)0.007 (<0.001)0.006 (<0.001)
F-box A protein	*fbxa-184*		11.7 (40.4)	0.005 (<0.001)
C-type lectin	*clec-190* *clec-223*	protein-interactions	245.0 (192.8)494.7 (177.8)	0.009 (<0.001)0.009 (0.004)
serpentine receptor, class A, class H, class I	*sra-14* *srh-85* *srh-43* *sri-40*	transmembrane receptorpseudogenepseudogenechemoreceptor	10.1 (16.5)152.3 (197.9)483.6 (104.1)30.1 (102.0)	<0.001 (<0.001)0.001 (0.003)<0.001 (0.002)0.004(<0.001)
protein kinase	*kin-34*	protein tyrosine kinase activity	11.6 (219.6)	0.006 (0.003)
C. elegans homeobox	*ceh-49* *ceh-83*	transcription factor activity	77.0 (166.0)17.1 (42.6)	0.007 (<0.001)0.006 (<0.001)
T box family	*tbx-9* *tbx-36*	transcription factor activity	37.6 (94.7)91.1 (98.8)	0.002 (<0.001)0.004 (<0.001)
regulator of G protein signaling	*rgs-8.1* *rgs-9*	signal transducer activity	17.9 (78.2)170.7 (192.2)	0.007 (0.002)0.002 (0.003)
FLYWCH zinc finger transcription factor homolog	*flh-3*	transcription factor activity	35.8 (97.2)	0.004 (<0.001)
laterally symmetric (defective in lateral symmetry)	*lsy-27*	transcription factor activity	114.5 (189.6)	0.007 (0.001)
patterned expression site	*pes-1* *pes-23*	transcription factor activitytransmembrane transporter activity	10.1 (34.4)97.2 (55.6)	0.004 (0.004)<0.001 (<0.001)
transthyretin-related family domain	*ttr-14* *ttr-34*	unknown	514.1 (144.2)140.3 (67.0)	0.002 (0.001)0.004 (<0.001)
maternal effect germ-cell defective	*meg-1*	P granule segregation	311.7 (159.3)	0.001 (0.001)
collagen	*col-135*	structural constituent of cuticle	779.4 (176.0)	0.003 (<0.001)
paired zinc finger protein	*pzf-1*	meiotic chromosome segregation	206.4 (127.8)	<0.001 (<0.001)
sperm specific family, class P	*ssp-37*	structural molecule activity	1013.1 (170.9)	<0.001 (<0.001)
zinc finger putative transcription factor family	*ztf-25*	transcription factor activity	257.4 (239.9)	0.001 (<0.001)
nanos related	*nos-2*	zinc ion binding	183.8 (161.5)	0.004 (<0.001)
trypsin-like protease	*try-1*	endopeptidase	93.6 (120.9)	0.004 (<0.001)
cell division cycle related	*cdc-25.3*	tyrosine phosphatase, cell cycle regulator	527.2 (173.9)	0.003 (<0.001)
acyltransferase-like	*acl-12*	acyltransferase activity	11.5 (28.2)	0.006 (<0.001)
eph(f)rin	*efn-3*	epidermal organization	33.8 (31.1)	<0.001 (<0.001)
FERM domain (protein4.1-ezrin-radixin-moesin) family	*frm-9*	unknown	52.5 (42.0)	0.002 (0.001)
RhoGAP for Rac-1 and cdc-42	*rrc-1*	signal transduction	58.2 (10.6)	0.006 (0.003)
regulator of fusion	*ref-1*	regulation of transcription	16.5 (28.0)	0.001 (<0.001)

a supplemental data contain a complete list of all hermaphrodite-specific genes (also unknown genes and genes without gene class name).

b sometimes the function of a gene is not precisely defined or only described as “predicted” function, information about genes was obtained from WormBase (WS229, www.wormbase.org).

Within the male- and hermaphrodite-specific genes, several transcription factor -encoding genes were differentially expressed between both sexes. The hermaphrodites exhibited much higher expression of *ceh-49* (adult: 166.0-fold, p<0.001), *ceh-83* (42.6-fold, p<0.001), *tbx-9* (94.7-fold, p<0.001), *tbx-36* (98.8, p<0.001), *flh-3* (97.2-fold, p<0.001), *lsy-27* (189.6-fold, p = 0.001), *pes-1* (34.4-fold, p = 0.004) and *ztf-25* (239.9-fold, p<0.001) (**Table 2**). In contrast, *mab-23* (43.5-fold, p<0.001), *mab-3* (44.0-fold, p = 0.003), *ceh-7* (11.2-fold, p<0.001), *nhr-266* (13.9-fold, p = 0.008) and *nhr-113* (72.5-fold, p = 0.002) were identified as male-specific transcription factors (**Table 1**). Moreover, some metabolic genes such as *lips-2*, *nas-17*, *nas-19* and *acl-12* were identified as being differentially expressed between males and hermaphrodites.

### Many C-type Lectin Genes are Expressed at Higher Levels in Males than in Hermaphrodites

**Figure 9:**
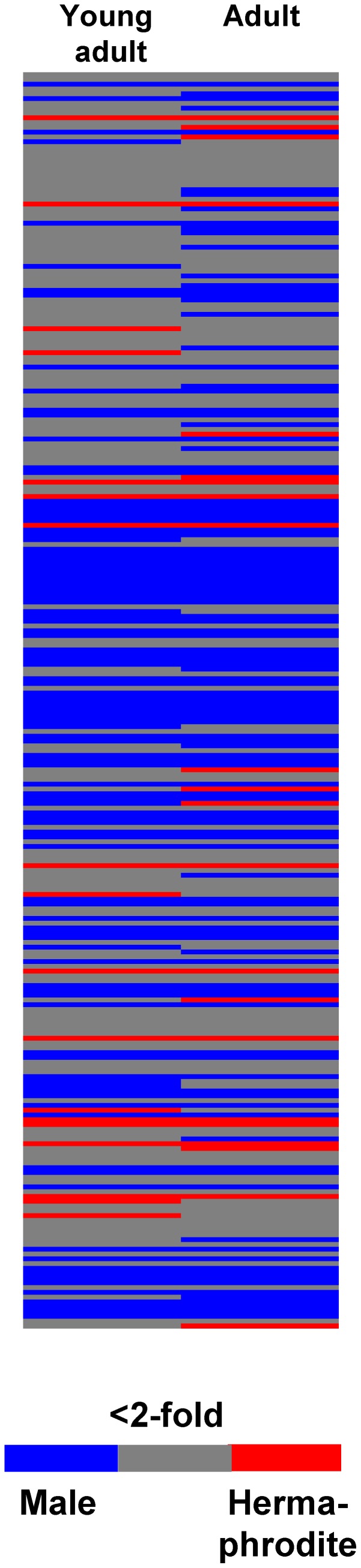
The differential expression of C-type lectin genes between *him-8 GFP* males and hermaphrodites at the young adulthood (66 h) and adulthood (76 h) stages. The differential expression level of each C-type lectin gene between *him-8 GFP* males and hermaphrodites is indicated using color. For red (blue), the expression level is at least 2-fold higher (p<0.05) in hermaphrodites (males) than in males (hermaphrodites). For items marked in grey, there is no difference in the expression level between both sexes. Each row shows one C-type lectin gene from *clec-1* (top) to *clec-266* (bottom).

Within the male-specific gene group (**Table 1**), many C-type lectin (*clec*) genes were expressed at significantly higher levels in males than in hermaphrodites. Notably, 42 *clec* genes exhibited male-specific gene expression, and only 2 *clec* genes were hermaphrodite-specific. Within the entire C-type lectin gene family, the male-enriched expression of *clec* genes was over-represented at both adult stages (**Figure 9**). More than 35% (95 genes) of all *clec* genes were expressed at higher levels (>2-fold, p<0.05) in males than in hermaphrodites. In comparison, less than 5% (12 genes) of all *clec* genes were expressed at higher levels in hermaphrodites than the males. Strikingly, many lectin-encoding genes were expressed at greater than 100-fold higher levels in males than in hermaphrodites.

### The Phosphofructokinase Gene C50F4.2 is Expressed at Much Higher Levels in Males than in Hermaphrodites

The observed differences in glucose and trehalose levels between males and hermaphrodites might be caused by differences in gene expression. Thus, we analyzed the expression of genes that encode enzymes involved in carbohydrate metabolism in both sexes (**Table 3**). Adult males expressed trehalose-6-phosphatase synthase (*tps-2*) at 2.68-fold (p<0.001) higher levels than did adult hermaphrodites, which confirms the trehalose measurements. The trehalose-6-phosphate phosphatase (*gob-1*) gene was also expressed at slightly higher levels in males (1.4/1.6 [two isoforms]). In contrast, adult hermaphrodites had higher mRNA levels of UTP-glucose-1-phosphate uridylyltransferase (2.5, p<0.01) and glycogen synthase (*gsy-1*, 2.2, p<0.01) than the adult males.

**Table 3:** Sex differences in the expression of carbohydrate metabolic genes^a^.

			Male to hermaphrodite ratio	
Gene ID	Gene name	Function^b^	Young adult	Adult
Trehalose metabolism				
ZK54.2b.1	*tps-1*	**trehalose-6-phosphatase synthase**(glucose-6-phosphatase+UDP-glucose trehalose 6-phosphate)	1.25	**1.17** *
ZK54.2a			**1.32** *	**1.23** *
F19H8.1	*tps-2*		**1.72** *	**2.68** ***
H13N06.3a	*gob-1*	**trehalose-6-phosphate phosphatase**(trehalose 6-phosphate trehalose)	1.04	1.36
H13N06.3b			1.15	**1.61** *
F57B10.7	*tre-1*	**trehalase**(trehalose 2×glucose)	**0.84** *	**0.71** **
T05A12.2.1	*tre-2*		**1.35** *	**1.45** **
T05A12.2.2			**1.53** *	**1.46** ***
W05E10.4	*tre-3*		1.11	**1.85** **
F15A2.2	*tre-4*		**0.96** *	0.99
C23H3.7	*tre-5*		1.15	1.18
Glycogen metabolism				
R05F9.6		**phosphotransferase**(glucose 6-phosphate glucose 1-phosphate)	0.94	**0.68** ***
Y43F4B.5a.1			0.98	0.95
Y43F4B.5a.2			0.97	**0.84** **
Y43F4B.5b			1.08	0.98
K08E3.5f		**UTP-glucose-1-phosphate uridylyltransferase**(glucose 1-phosphate UDP-glucose)	**0.66** **	**0.39** **
K08E3.5a.1			**0.71** *	**0.41** ***
Y46G5A.31	*gsy-1*	**glycogensynthase**(UDP-glucose glycogen)	0.87	**0.46** **
T22F3.3a		**glycogen phosporylase**(glycogen glucose 1-phosphate)	0.85	**0.53** **
T22F3.3b.2			0.93	**0.51** ***
Glucose metabolism				
F14B4.2a.2		**hexokinase**(glucose glucose 6-phosphate)	0.79	**0.43** **
F14B4.2b			**0.60** *	**0.28** ***
Y87G2A.8b	*gpi-2*	**Glucose-6-phosphate isomerase**(glucose 6-phosphate fructose 6-phophate)	0.92	**0.78** **
Y87G2A.8a.2			0.96	0.89
C50F4.2		**6-phosphofructokinase**(fructose-6P fructose -1,6 bisphosphate)	**3.33** ***	**29.6** **
Y71H10A.1a			**1.33** ***	**1.61** ***
Y71H10A.1b.3			**1.54** *	**1.83** *
Y71H10A.1b.1			1.10	0.98
T05D4.1.2	*aldo-1*	**fructose bisphosphate aldolase**(fructose-1,6 bisphosphate glyceraldehyd-3 phosphate)	**2.11** *	**2.85** ***
T05D4.1.1			**2.12** *	**2.33** **
F01F1.12a	*aldo-2*		0.94	0.81
F01F1.12b.1			0.99	0.94
F33H1.2.2	*gpd-4*	**Glyceraldehyd-3-phosphate dehydrogenase**(glyceraldehyd-3 phosphate glycerate-1,3 bisphosphate)	**0.38** **	**0.19** ***
F33H1.2.1			**0.39** *	**0.20** **
K10B3.7.2	*gpd-3*		1.00	0.96
T09F3.3.1	*gpd-2*		**0.37** **	**0.19** **
T09F3.3.2			**0.45** **	**0.23** ***
T03F1.3	*pgk-1*	**phosphoglycerate kinase**(glycerate-1,3 bisphosphate glycerate-3 phosphate)	0.89	**0.69** ***
F57B10.3b.2		**phosphoglycerate mutase** (glycerate-3 phosphate glycerate-2 phosphate)	1.03	0.99
F57B10.3a			1.05	0.97
T21B10.2a.1	*enol-1*	**enolase**(glycerate-2 phosphate phosphoenolpyruvate)	**0.90** *	**0.79** **
T21B10.2b.1			0.92	**0.82** *
F25H5.3a	*pyk-1*	**pyruvate kinase** (phosphoenolpyruvate pyruvate)	0.91	**0.79** *
F25H5.3e			1.18	**2.17** **
ZK593.1	*pyk-2*		**0.68** *	**1.16** *

a Asterisks indicate statistical significance measured by t-test,

* p<0.05,

** p<0.01,

*** p<0.001, blue (red): significantly expressed in males (hermaphrodites).

b Genes, which are involved in carbohydrate metabolism, were identified with KEGG pathway (www.genome.jp/kegg).

Consistent with the differences observed in trehalose and glycogen metabolism, sex differences were also found in the expression of genes encoding glycolytic enzymes. Hexokinase (2.3/3.5 [two isoforms], p<0.01, **Table 3**) was expressed at higher levels in adult hermaphrodites than in adult males. In contrast, adult males expressed phosphofructokinase and pyruvate kinase at higher levels. In *C. elegans,* two genes, C50F4.2 and Y71H10A.1, encode putative phosphofructokinases. Both genes were expressed at higher levels in the adult males. Remarkably, the expression of C50F4.2 was more than 29-fold (p = 0.004) higher in the males than in the hermaphrodites. Additionally, one homolog of fructose bisphosphate aldolase (T05D4.1) was expressed at higher levels in the adult males than in the adult hermaphrodites (2.3/2.8 [two isoforms], p<0.01). Taken together, these data imply that glycolysis and trehalose synthesis may be upregulated in males, resulting in lower glucose and equal or higher trehalose levels compared to the hermaphrodites.

## Discussion

The nematode *C. elegans* is one of the most widely used model organisms in modern biology. Recently, *C. elegans* has also been established as a model to examine physiological aspects at the molecular and cellular levels in an intact organism [39]. Thus far, sex differences in physiology and metabolism have not been studied extensively in *C. elegans* because it is laborious to separately analyze large numbers of males and hermaphrodites. To circumvent this methodological problem, we used male-enriched mutants, which included one mutant with a sex-specific GFP signal. In all analyses, males and hermaphrodites were cultivated on the same plate and separated using flow cytometry. Using this approach, we found that the adult males exhibited smaller body size, nearly equal triglyceride and protein levels, lower total RNA content, higher trehalose levels and lower glucose levels than the hermaphrodites. These differences were also present when the values were adjusted for body volume. We also identified a number of genes that were expressed differentially between the sexes, and this finding may explain some physiological differences between the *C. elegans* adult males and hermaphrodites.

### Adult Males are Smaller and Contain Lower RNA Content than Hermaphrodites but have Similar Body Composition

Before and at the young adult stage (66 h), only minor differences were observed between the males and hermaphrodites regarding morphology, body proportion and body composition. These results are consistent with the findings of Thoemke *et al.*, who found few differences at the transcriptional level between males and hermaphrodites up to the L4 larval stage [24]. At the young adult and adult stages, the total RNA content is significantly lower in males than in hermaphrodites. The total RNA content did not change from the young adult to the adult stage in males, whereas the total RNA content of hermaphrodites increased considerably during this period. Total RNA is mainly composed of ribosomal RNA (rRNA, visible in RNA electrophoretic analysis) followed by transfer RNA (tRNA), mRNA and a small amount of non-coding RNA [40]. Adult hermaphrodites transcribe many RNAs and incorporate them into their developing oocytes. Thus, hermaphrodites may have a much higher RNA content due to embryogenesis. Males are more than 50% smaller than hermaphrodites. Recent work by Honjoh *et al.* demonstrated that inhibition of the translation machinery reduces body length to a greater extent in hermaphrodites than in males (personal communication, S. Honjoh, [41]). The authors assumed that males have a lower translation rate. Thus, the lower growth rate in males might be caused by a lower translation rate and thereby, a lower rate of protein biosynthesis. The triglyceride-to-protein ratio and the volume-adjusted triglyceride and protein levels were similar between males and hermaphrodites. Therefore, sex differences in body composition, as described in other organisms [42]–[45], are not obvious in *C. elegans*. Notably, the determined protein and triglyceride values for adult hermaphrodites are similar to literature values [27]–[29]. Thus, we found sex differences in body proportions and RNA content but not in body composition.

### Carbohydrate Metabolism Differs between the *C. elegans* Sexes at the Metabolite and Gene Expression Level

**Figure 10:**
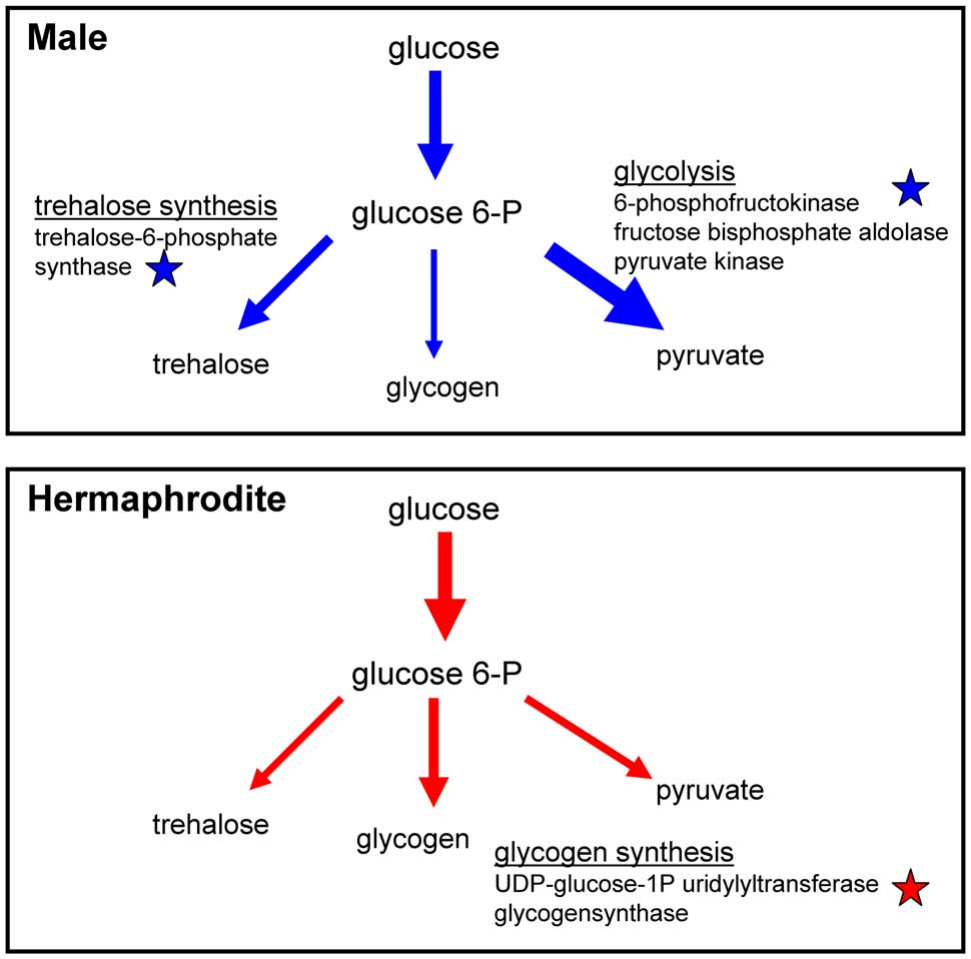
The conversion of glucose in males and hermaphrodites. Glucose can be catabolized to pyruvate in glycolysis converted to trehalose or synthesized to glycogen. How pronounced each pathway is in each sex is indicated by the thickness of the arrows. Asterisks indicate significantly regulated genes.

Trehalose is a soluble, non-reducing disaccharide comprising two molecules of glucose. This molecule is present in many bacteria, yeast and invertebrates [30], [31] and is synthesized in a two-step pathway from glucose-6-phosphate and UDP-glucose by trehalose-6-phosphate synthase and trehalose-6-phosphate phosphatase [30]. Two trehalose phosphate synthase (*tps-1* and *tps-2*) and one trehalose phosphate phosphatase (*gob-1*) genes have been characterized in *C. elegans* [46], [47]. Both of the substrates of trehalose synthesis are also needed for glycogen synthesis. Based on our results, we hypothesized that males utilize glucose (UDP-glucose, glucose-6-phosphate) to produce trehalose rather than glycogen; the opposite may be true in hermaphrodites (**Figure 10**). This hypothesis is reflected by gene expression data demonstrating higher expression of trehalose synthesis genes and lower expression of glycogen synthesis genes in males than in hermaphrodites (**Table 3**). Also in agreement with this hypothesis, we found higher trehalose levels in males than in hermaphrodites. In our experiments, higher glucose than trehalose levels were measured in males and hermaphrodites, which is contrary to other reports [30], [32]. A reason for this discrepancy could lie in the different methods used. In our study, the biochemical results were validated using NMR spectroscopy, and similar levels could be measured in different mutants.

Evidence from several studies indicates that trehalose has various functions in the organism [31]. In *C. elegans,* it is believed that trehalose serves as a sugar transporter between cell types because a functional glucose-6-phophatase is lacking [30], [31], [33]. It has also been predicted that trehalose protects worms and other nematodes against environmental stresses including desiccation, heat and hypertonia [30], [38], [48] by protecting lipid membranes and stabilizing protein structures [49], [50]. Additionally, trehalose extends the lifespan of *C. elegans* [37], which is most likely caused by the trehalose-mediated prevention of age-related protein damage. Eggs and dauer larvae, which are highly resistant against environmental stresses, contain high levels of trehalose [30], [47]. The higher levels of trehalose in males could mean that males are more resistant against desiccation and heat stress than hermaphrodites and age more slowly. Whether this is true will need to be clarified by further studies.

A much lower glucose level was found in males than in hermaphrodites. This finding was supported by the gene expression data reported here because genes encoding glycolytic enzymes were more highly expressed in males than in hermaphrodites. Importantly, the key enzyme of glycolysis, phosphofructokinase, was expressed at a 29-fold higher level in males. This gene also exhibited male-enriched gene expression in other microarray studies [22], [23]. Thus, these data suggest that males exhibit higher flux through glycolysis than do hermaphrodites (**Figure 10**). Based on mammalian studies, it is known that glycolysis-encoding genes are extensively modified during spermatogenesis [51] because sperm require large amounts of ATP for motility [52]–[54]. Furthermore, spermiogenesis is blocked by mitochondrial inhibitors in *C. elegans*, which indicates that aerobic metabolism, and therefore glycolysis, may be essential for sperm production [55]. All of these findings suggest that male *C. elegans* have higher glycolysis rates due to spermatogenesis and spermiogenesis. This hypothesis is further supported by the fact that phosphofructokinase (C50F4.2) transcripts are enriched during spermatogenesis [23].

### Lectin Genes are Differentially Expressed between the *C. elegans* Sexes

In microarray experiments, male- and hermaphrodite-specific genes were identified. Notably, hermaphrodites and males were grown under the same conditions on the same plate. Thus far, all studies analyzing sex-specific gene expression did not consider these criteria [22]–[24]. Nevertheless, there is a high degree of agreement between the published data [22]–[24] and the data presented here. In at least one of these studies, 76.2% of male-specific genes have been described as male-specific genes. At least two of these studies have described 43.9% of these genes. For hermaphrodite-specific genes, a similar agreement was found (80.3% and 43.4%, respectively). Surprisingly, few metabolic genes exhibiting a sex-specific pattern (*lips-2*, *nas-17*, *nas-19* and *acl-12*) were detected. It can be concluded that males and hermaphrodites possess fundamentally similar pathways in intermediary metabolism. Instead, quantitative sex differences in metabolism, as found here for carbohydrate metabolism, can be expected.

Among the sex-specific genes examined, many transcription factors were differentially expressed in both sexes. All of the hermaphrodite-specific transcription factors that were identified function in embryogenesis [56]–[59]; hence, no reproduction-independent transcription factor was found. The male-specific transcription factors were either involved in the development of the tail or male-specific neurons [60]–[63] or the function is unknown. All of the male-specific transcription factors identified here have been previously described as enriched in males [22]. Thus, no new transcription factors involved in sex differentiation in *C. elegans* were discovered.

Many C-type lectin genes were expressed at dramatically higher levels in males than in hermaphrodites, and 42 of these genes were classed as male-specific. In accordance with this finding, 30 of these genes were designated as male-enriched in previous sex-specific gene expression studies [22], [23]. C-type lectins are a diverse group of proteins, which are currently represented by 265 genes. These lectins occur in plants, bacteria, viruses and animals as a class of proteins involved in various protein interactions [64]. Some members of this gene family appear to be involved in innate immunity because they are upregulated after pathogen exposure [65]. However, the functional role of C-type lectins in invertebrates is essentially unknown. The sex-specific expression of C-type lectins could be explained by either of two models. The first model suggests that the immune system is sexually dimorphic. This is confirmed by the fact that defense-related genes other than C-type lectins are also differentially expressed between males and hermaphrodites (**Table 1**). In rodents and *C. elegans*, sex-specific survival after pathogenic infections has been observed [21], [66]. The second model assumes that C-type lectins fulfill sex-specific tasks in males. In line with this hypothesis, only three out of 42 male-specific C-type lectin genes (*clec-217*, *clec-256* and *clec-263*) were found to be upregulated after pathogen infection [65]. In contrast, several *clec* genes (*clec-197*, *clec-99*, *clec-92*, *clec-157*, *clec-208* and *clec-207*) and *scl-8*, which were identified as male-specific or male-enriched in our analysis, are associated with male sperm chromatin [67]; this finding supports their putative role in spermatogenesis. Furthermore, one study has shown that C-type lectins are regulated by *mxl-2* (max-like protein, a bHLHZip transcription factor), which is involved in cell migration [68]. Further investigation, especially regarding the natural function of this gene family, is needed to confirm that these C-type lectins fulfill special roles in male *C. elegans*.

Taken together, this study combines the use of a male mutant expressing a sex-specific GFP signal with flow cytometry to analyze a large number of male and hermaphrodite *C. elegans* individuals under standardized conditions. In comparison to adult hermaphrodites, males are smaller and contain less RNA but have similar fat to fat-free mass ratios. Males also exhibit higher trehalose and lower glucose levels than hermaphrodites. These sex differences are linked to the expression levels of genes that encode key enzymes in carbohydrate metabolism. Moreover, certain lectin genes were identified as being differentially expressed between the *C. elegans* sexes. Thus, physiological differences between male and hermaphrodite *C. elegans* individuals are linked to gene expression.

## Materials and Methods

### Ethics Statement

N/A.

### Nematode Cultivating and Strains

*C. elegans* worms were maintained on NGM agar plates seeded with *E. coli* OP50 at 20°C as described by Brenner [69]. The strain Bristol N2 was used as the wild type control. The mutants used in this study were *fog-2 (q71)* V, *him-8 (e1489)* IV, *him-5 (e1490)* V and *him-8 (e1489)* IV; *nls128* II [pkd-2::GFP; lin-15 (+)]. All strains were obtained from the *Caenorhabditis* Genetics Center (CGC, University of Minnesota, USA) except for *him-8 (e1489)* IV; *nls128* II, which was generously provided by the Horvitz laboratory. All experiments were carried out with synchronized eggs obtained by hypochlorite treatment of gravid worms. The plate-based dietary restriction method was established in our laboratory by D. Palgunow.

### Microscopic Imaging and Determination of Body Proportions

Worms were visualized using an Axio Observer D1 inverted microscope (Zeiss, Jena, Germany) equipped with a digital camera (Axiocam MRm, Zeiss). Body length, width, perimeter and area were determined by bordering the worms in Axio Vision Software (version 4.8, Zeiss). Body volume was determined from the area and perimeter [70], [71].

### COPAS Flow Cytometric Analysis

Flow cytometry was used to select a defined number of eggs for equal experimental settings, to analyze worm characteristics and to separate males from hermaphrodites to prepare worm extracts and isolate RNA. Axial length (Time of Flight, TOF), optical density (Extinction, EXT) and fluorescence (Green, Yellow, Red) were automatically measured for each worm. The instrument was equipped as described by Klapper *et al.* [27]. The gain signals for extinction and green, yellow and red fluorescence were set to 1. The photomultiplier (PMT) value for the green channel was set to 900. The threshold value for signals and the TOF minimum were adopted depending on whether eggs or worms were sorted. For all experiments, specific gating and sorting criteria were used.

### Separation of Males Using COPAS Flow Cytometry

The separation procedure used to generate pure male samples depended on the strain used. The transgenic strain *him-8 (e1489)* IV; *nls128* II expresses GFP in male-specific neurons under the control of the *pkd-2* promoter. Thus, males were separated from hermaphrodites based on a specific green fluorescence signal using flow cytometry. No separation was possible before the young adult stage (approximately 64–66 h) due to low signal strength during the earlier stages. Based on this mutant strain, it was possible to obtain almost 100% males. For N2 and all other mutants (*fog-2*, *him-8*, and *him-5*), the separation of males was achieved by choosing an area in green versus yellow fluorescence (auto-fluorescence) of a dot blot because hermaphrodites exhibit a higher auto-fluorescence than males. Because good separation rates were only attained when worms had reached one day of adulthood (90 h), all measurements were carried out at this time point. The rate of males ranged between 87% and 99% depending on the strain.

### Worm Extracts and Biochemical Assays

Using flow cytometry, 1000–1500 worms were collected in 2 ml tubes suspended in 100 µl NET buffer (50 mM Tris pH 7.5, 150 mM NaCl, 1 mM EDTA pH 8.0, 0.5% CHAPS, protease inhibitor cocktail [Roche Diagnostics GmbH, Mannheim, Germany]) and homogenized using a bead-beating homogenizer (Precellys®24, Bertin Technologies, Montigny le Bretonneux, France). Worm extracts were made using ceramic beads (1.4 mm) at full speed (6500 rpm) for 20 s as 1 cycle. The cell debris was pelleted at full speed for 20 min at 4°C and then removed. The resulting supernatants were stored at −80°C until analyzed in the biochemical assays. The protein and triglyceride content was measured in worm extracts using the BCA Protein assay kit (Pierce Protein assay kit, Rockford, USA) and an enzymatic triglyceride assay kit (Analyticon, Lichtenfels, Germany) with a separate triacylglycerol standard (BioCat, Heidelberg, Germany), respectively. Trehalose and glucose were quantified simultaneously using a trehalose assay kit (Megazyme, Ireland). TOF and EXT values were also measured during worm sorting. To equalize body volume differences between males and hermaphrodites, a hermaphrodite-to-male extinction ratio was calculated, and this was used to normalize male body composition values.

### NMR Spectroscopy

Five thousand worms per sample were collected using flow cytometry and homogenized in 270 µl distilled water by Precellys®24 under the same conditions as those mentioned above. Undissolved material was removed by centrifugation and the samples were stored at −80°C. NMR analysis was carried out at LipoFIT Analytic GmbH (Regensburg, Germany) on a Bruker Avance II + operating at 600 MHz. The amounts of glucose and trehalose were determined by normalizing each integral to the corresponding integral of the standard solution. Each experiment was performed in triplicate. The same extracts were also analyzed biochemically and compared with measurements performed with NET buffer.

### Isolation of Total RNA for Gene Expression Analysis

Between 1200 and 1800 worms were collected using flow cytometry and suspended in 350 µl RLT buffer (RNeasy mini Kit, Qiagen, Hilden, Germany). The worms were then homogenized with Precellys®24 (full speed, 15 s, 2 cycles) and QIAshredder columns (Qiagen). Total RNA was extracted using an RNeasy mini Kit (Qiagen), which included a DNA digestion step according to the manufacturer’s instructions. The amount and integrity of the RNA was assessed spectrophotometrically (BioPhotometer, Eppendorf, Hamburg, Germany) and using a Bioanalyzer 2100 (Agilent Technologies, Santa Clara, USA) and the RNA 6000 Nano Kit (Agilent Technologies). Each sample contained at least 10 µg of total RNA per µl.

### Gene Expression Analysis

Microarray processing and the normalization of row data were performed by Source Bioscience (imaGenes, Berlin, Germany) using a custom-designed Agilent gene expression microarray. This microarray was developed by imaGenes/Source Bioscience (Steffen Hennig) and contained 61643 oligonucleotides, which resulted in 26843 genes. Quantile normalization was calculated using R-package [72]. Each experiment was performed in triplicate. To analyze sex differences, the average expression of each gene with a sex-specific ratio (male- or hermaphrodite-specific) was calculated. A statistical cut-off of p<0.01 (Student’s two-tailed *t*-test) was applied. Gene ontology of male- and hermaphrodite-specific genes was analyzed with the WormBase database (WormBase release WS229). Genes involved in carbohydrate metabolism were identified using the Kyoto Encyclopedia of Genes and Genomes pathway (KEGG, http://www.genome.jp/kegg/).

### Statistical Analysis

The data are expressed as the mean ± standard deviations (SDs). To determine statistically significant differences among the tested male strains, one-way analysis of variance (ANOVA) was performed, and this was followed by the Bonferroni test (**Figure 4**). When an inhomogeneity of variance was evident, Dunnett’s T3 test was used as a post hoc test. Correlations between TOF and body length and between EXT and body volume were determined using linear regression with calculation of the coefficients of determination (r^2^) and p values (**Figure 2**). For all other results, the Student’s *t*-test was used as a statistical test for the consideration of variance homogeneity. The results were classed as significant at p<0.05. All statistical analysis was performed using SPSS software (Statistical Package for the Social Sciences, version 11.0).

## Supporting Information

Table S1 **Body composition of males and hermaphrodites at different developmental stages in different male mutants.**
(DOC)

Table S2 **Glucose and trehalose content of males and hermaphrodites at different developmental stages in different male mutants.**
(DOC)

Table S3 **The entire list of male-specific genes.**
(DOC)

Table S4 **The entire list of hermaphrodite-specific genes.**
(DOC)

## References

[1] Hodgkin J, Horvitz HR, Brenner S (1979) Nondisjunction Mutants of the Nematode CAENORHABDITIS ELEGANS. Genetics 91: 67–94. 17248881 10.1093/genetics/91.1.67 PMC1213932

[2] LaMunyon CW, Ward S (1998) Larger sperm outcompete smaller sperm in the nematode Caenorhabditis elegans. Proc Biol Sci 265: 1997–2002. 9821364 10.1098/rspb.1998.0531 PMC1689481

[3] Ward S, Carrel JS (1979) Fertilization and sperm competition in the nematode Caenorhabditis elegans. Dev Biol 73: 304–321. 499670 10.1016/0012-1606(79)90069-1

[4] Sulston JE, Albertson DG, Thomson JN (1980) The Caenorhabditis elegans male: postembryonic development of nongonadal structures. Dev Biol 78: 542–576. 7409314 10.1016/0012-1606(80)90352-8

[5] Sulston JE, Horvitz HR (1977) Post-embryonic cell lineages of the nematode, Caenorhabditis elegans. Dev Biol 56: 110–156. 838129 10.1016/0012-1606(77)90158-0

[6] Kimble J, Hirsh D (1979) The postembryonic cell lineages of the hermaphrodite and male gonads in Caenorhabditis elegans. Dev Biol 70: 396–417. 478167 10.1016/0012-1606(79)90035-6

[7] Emmons SW, Sternberg PW (1997) Male Development and Mating Behavior. in C. elegans II: Chapter 12, 295–334. 21413228

[8] Hodgkin J (1988) Sexual dimorphism and sex determination. In the nematode C elegans (ed WB Wood) Chap. 9: 243–279.

[9] Portman DS (2007) Genetic control of sex differences in C. elegans neurobiology and behavior. Adv Genet 59: 1–37. 17888793 10.1016/S0065-2660(07)59001-2

[10] Hardaker LA, Singer E, Kerr R, Zhou G, Schafer WR (2001) Serotonin modulates locomotory behavior and coordinates egg-laying and movement in Caenorhabditis elegans. J Neurobiol 49: 303–313. 11745666 10.1002/neu.10014

[11] Liu KS, Sternberg PW (1995) Sensory regulation of male mating behavior in Caenorhabditis elegans. Neuron 14: 79–89. 7826644 10.1016/0896-6273(95)90242-2

[12] Loer CM, Kenyon CJ (1993) Serotonin-deficient mutants and male mating behavior in the nematode Caenorhabditis elegans. J Neurosci 13: 5407–5417. 8254383 10.1523/JNEUROSCI.13-12-05407.1993 PMC6576401

[13] Lee K, Portman DS (2007) Neural sex modifies the function of a C. elegans sensory circuit. Curr Biol 17: 1858–1863. 17964163 10.1016/j.cub.2007.10.015

[14] Lipton J, Kleemann G, Ghosh R, Lints R, Emmons SW (2004) Mate searching in Caenorhabditis elegans: a genetic model for sex drive in a simple invertebrate. J Neurosci 24: 7427–7434. 15329389 10.1523/JNEUROSCI.1746-04.2004 PMC6729642

[15] Mah KB, Rankin CH (1992) An analysis of behavioral plasticity in male Caenorhabditis elegans. Behav Neural Biol 58: 211–221. 1456943 10.1016/0163-1047(92)90496-q

[16] Vellai T, McCulloch D, Gems D, Kovacs AL (2006) Effects of sex and insulin/insulin-like growth factor-1 signaling on performance in an associative learning paradigm in Caenorhabditis elegans. Genetics 174: 309–316. 16849598 10.1534/genetics.106.061499 PMC1569791

[17] Smith DW (1989) Is greater female longevity a general finding among animals? Biol Rev Camb Philos Soc 64: 1–12. 2655725 10.1111/j.1469-185x.1989.tb00635.x

[18] Gems D, Riddle DL (2000) Genetic, behavioral and environmental determinants of male longevity in Caenorhabditis elegans. Genetics 154: 1597–1610. 10747056 10.1093/genetics/154.4.1597 PMC1461011

[19] Gems D, Riddle DL (1996) Longevity in Caenorhabditis elegans reduced by mating but not gamete production. Nature 379: 723–725. 8602217 10.1038/379723a0

[20] Inoue H, Nishida E (2010) The DM domain transcription factor MAB-3 regulates male hypersensitivity to oxidative stress in Caenorhabditis elegans. Mol Cell Biol 30: 3453–3459. 20498281 10.1128/MCB.01459-09 PMC2897546

[21] van den Berg MC, Woerlee JZ, Ma H, May RC (2006) Sex-dependent resistance to the pathogenic fungus Cryptococcus neoformans. Genetics 173: 677–683. 16582430 10.1534/genetics.106.056093 PMC1526500

[22] Jiang M, Ryu J, Kiraly M, Duke K, Reinke V, et al (2001) Genome-wide analysis of developmental and sex-regulated gene expression profiles in Caenorhabditis elegans. Proc Natl Acad Sci U S A 98: 218–223. 11134517 10.1073/pnas.011520898 PMC14571

[23] Reinke V, Gil IS, Ward S, Kazmer K (2004) Genome-wide germline-enriched and sex-biased expression profiles in Caenorhabditis elegans. Development 131: 311–323. 14668411 10.1242/dev.00914

[24] Thoemke K, Yi W, Ross JM, Kim S, Reinke V, et al (2005) Genome-wide analysis of sex-enriched gene expression during C. elegans larval development. Dev Biol 284: 500–508. 15987632 10.1016/j.ydbio.2005.05.017

[25] Hirose T, Nakano Y, Nagamatsu Y, Misumi T, Ohta H, et al (2003) Cyclic GMP-dependent protein kinase EGL-4 controls body size and lifespan in C elegans. Development 130: 1089–1099. 12571101 10.1242/dev.00330

[26] Jonassen T, Marbois BN, Faull KF, Clarke CF, Larsen PL (2002) Development and fertility in Caenorhabditis elegans clk-1 mutants depend upon transport of dietary coenzyme Q8 to mitochondria. J Biol Chem 277: 45020–45027. 12324451 10.1074/jbc.M204758200

[27] Klapper M, Ehmke M, Palgunow D, Bohme M, Matthaus C, et al (2011) Fluorescence-based fixative and vital staining of lipid droplets in Caenorhabditis elegans reveal fat stores using microscopy and flow cytometry approaches. J Lipid Res 52: 1281–1293. 21421847 10.1194/jlr.D011940 PMC3090249

[28] Takahashi K, Yoshina S, Masashi M, Ito W, Inoue T, et al (2009) Nematode homologue of PQBP1, a mental retardation causative gene, is involved in lipid metabolism. PLoS One 4: e4104. 19119319 10.1371/journal.pone.0004104 PMC2606030

[29] Zarse K, Ristow M (2008) Antidepressants of the serotonin-antagonist type increase body fat and decrease lifespan of adult Caenorhabditis elegans. PLoS One 3: e4062. 19112515 10.1371/journal.pone.0004062 PMC2605556

[30] Behm CA (1997) The role of trehalose in the physiology of nematodes. Int J Parasitol 27: 215–229. 9088992 10.1016/s0020-7519(96)00151-8

[31] Elbein AD, Pan YT, Pastuszak I, Carroll D (2003) New insights on trehalose: a multifunctional molecule. Glycobiology 13: 17R–27R. 10.1093/glycob/cwg047 12626396

[32] Hanover JA, Forsythe ME, Hennessey PT, Brodigan TM, Love DC, et al (2005) A Caenorhabditis elegans model of insulin resistance: altered macronutrient storage and dauer formation in an OGT-1 knockout. Proc Natl Acad Sci U S A 102: 11266–11271. 16051707 10.1073/pnas.0408771102 PMC1183534

[33] McElwee JJ, Schuster E, Blanc E, Thomas JH, Gems D (2004) Shared transcriptional signature in Caenorhabditis elegans Dauer larvae and long-lived daf-2 mutants implicates detoxification system in longevity assurance. J Biol Chem 279: 44533–44543. 15308663 10.1074/jbc.M406207200

[34] Phillips CM, Wong C, Bhalla N, Carlton PM, Weiser P, et al (2005) HIM-8 binds to the X chromosome pairing center and mediates chromosome-specific meiotic synapsis. Cell 123: 1051–1063. 16360035 10.1016/j.cell.2005.09.035 PMC4435792

[35] Barr MM, Sternberg PW (1999) A polycystic kidney-disease gene homologue required for male mating behaviour in C. elegans. Nature 401: 386–389. 10517638 10.1038/43913

[36] Schedl T, Kimble J (1988) fog-2, a germ-line-specific sex determination gene required for hermaphrodite spermatogenesis in Caenorhabditis elegans. Genetics 119: 43–61. 3396865 10.1093/genetics/119.1.43 PMC1203344

[37] Honda Y, Tanaka M, Honda S (2010) Trehalose extends longevity in the nematode Caenorhabditis elegans. Aging Cell 9: 558–569. 20477758 10.1111/j.1474-9726.2010.00582.x

[38] Lamitina ST, Strange K (2005) Transcriptional targets of DAF-16 insulin signaling pathway protect C. elegans from extreme hypertonic stress. Am J Physiol Cell Physiol 288: C467–474. 15496475 10.1152/ajpcell.00451.2004

[39] Jones KT, Ashrafi K (2009) Caenorhabditis elegans as an emerging model for studying the basic biology of obesity. Dis Model Mech 2: 224–229. 19407330 10.1242/dmm.001933 PMC2675801

[40] Lindberg J, Lundeberg J The plasticity of the mammalian transcriptome. Genomics 95: 1–6. 19716875 10.1016/j.ygeno.2009.08.010

[41] Honjoh S, Kajiwara Y, Nishida E (2011) The sexual dimorphic response to dietary restriction. C elegans meeting 2011 Abstract volume, Abstract number 256B: 78.

[42] Bonnet X, Shine R, Naulleau G, Vacher-Vallas M (1998) Sexual dimorphism in snakes: different reproductive roles favour different body plans. Proc R Soc Lond B 265: 179–183.

[43] Boos M, Zorn T, Le Maho Y, Groscolas R, Robin JP (2002) Sex differences in body composition of wintering Mallards (Anas platyrhynchos): possible implications for survival and reproductive performance. Bird Study 49: 212–218.

[44] Kirchengast S (2010) Gender Differences in body composition from childhood to old age: an evolutionary point of view. J Life Sci 2: 1–10.

[45] Schulte-Hostedde AI, Millar JS, Hickling GJ (2001) Sexual dimorphism in body composition of small mammals. Can J Zool 79: 1016–1020.

[46] Kormish JD, McGhee JD (2005) The C. elegans lethal gut-obstructed gob-1 gene is trehalose-6-phosphate phosphatase. Dev Biol 287: 35–47. 16197937 10.1016/j.ydbio.2005.08.027

[47] Pellerone FI, Archer SK, Behm CA, Grant WN, Lacey MJ, et al (2003) Trehalose metabolism genes in Caenorhabditis elegans and filarial nematodes. Int J Parasitol 33: 1195–1206. 13678635 10.1016/s0020-7519(03)00173-5

[48] Jagdale GB, Grewal PS, Salminen SO (2005) Both heat-shock and cold-shock influence trehalose metabolism in an entomopathogenic nematode. J Parasitol 91: 988–994. 16419738 10.1645/GE-504R.1

[49] Crowe JH, Crowe LM (2000) Preservation of mammalian cells-learning nature’s tricks. Nat Biotechnol 18: 145–146. 10657114 10.1038/72580

[50] Jain NK, Roy I (2009) Effect of trehalose on protein structure. Protein Sci 18: 24–36. 19177348 10.1002/pro.3 PMC2708026

[51] Vemuganti SA, de Villena FP, O’Brien DA (2010) Frequent and recent retrotransposition of orthologous genes plays a role in the evolution of sperm glycolytic enzymes. BMC Genomics 11: 285. 20459611 10.1186/1471-2164-11-285 PMC2881024

[52] Miki K, Qu W, Goulding EH, Willis WD, Bunch DO, et al (2004) Glyceraldehyde 3-phosphate dehydrogenase-S, a sperm-specific glycolytic enzyme, is required for sperm motility and male fertility. Proc Natl Acad Sci U S A 101: 16501–16506. 15546993 10.1073/pnas.0407708101 PMC534542

[53] Mukai C, Okuno M (2004) Glycolysis plays a major role for adenosine triphosphate supplementation in mouse sperm flagellar movement. Biol Reprod 71: 540–547. 15084484 10.1095/biolreprod.103.026054

[54] Peterson RN, Freund M (1969) Glycolysis by washed suspensions of human spermatozoa. Effect of substrate, substrate concentration, and changes in medium composition on the rate of glycolysis. Biol Reprod 1: 238–246. 5406653 10.1095/biolreprod1.3.238

[55] Ward S, Hogan E, Nelson GA (1983) The initiation of spermiogenesis in the nematode Caenorhabditis elegans. Dev Biol 98: 70–79. 6345236 10.1016/0012-1606(83)90336-6

[56] Andachi Y (2004) Caenorhabditis elegans T-box genes tbx-9 and tbx-8 are required for formation of hypodermis and body-wall muscle in embryogenesis. Genes Cells 9: 331–344. 15066124 10.1111/j.1356-9597.2004.00725.x

[57] Baugh LR, Hill AA, Slonim DK, Brown EL, Hunter CP (2003) Composition and dynamics of the Caenorhabditis elegans early embryonic transcriptome. Development 130: 889–900. 12538516 10.1242/dev.00302

[58] Hope IA (1994) PES-1 is expressed during early embryogenesis in Caenorhabditis elegans and has homology to the fork head family of transcription factors. Development 120: 505–514. 8162851 10.1242/dev.120.3.505

[59] Zhang F, O’Meara MM, Hobert O (2011) A left/right asymmetric neuronal differentiation program is controlled by the Caenorhabditis elegans lsy-27 zinc-finger transcription factor. Genetics 188: 753–759. 21555395 10.1534/genetics.111.129064 PMC3176537

[60] Kagoshima H, Cassata G, Burglin TR (1999) A Caenorhabditis elegans homeobox gene expressed in the male tail, a link between pattern formation and sexual dimorphism? Dev Genes Evol 209: 59–62. 9914419 10.1007/s004270050227

[61] Lints R, Emmons SW (2002) Regulation of sex-specific differentiation and mating behavior in C. elegans by a new member of the DM domain transcription factor family. Genes Dev 16: 2390–2402. 12231628 10.1101/gad.1012602 PMC187445

[62] Raymond CS, Shamu CE, Shen MM, Seifert KJ, Hirsch B, et al (1998) Evidence for evolutionary conservation of sex-determining genes. Nature 391: 691–695. 9490411 10.1038/35618

[63] Shen MM, Hodgkin J (1988) mab-3, a gene required for sex-specific yolk protein expression and a male-specific lineage in C. elegans. Cell 54: 1019–1031. 3046751 10.1016/0092-8674(88)90117-1

[64] Kilpatrick DC (2002) Animal lectins: a historical introduction and overview. Biochim Biophys Acta 1572: 187–197. 12223269 10.1016/s0304-4165(02)00308-2

[65] Schulenburg H, Hoeppner MP, Weiner J, 3rd, Bornberg-Bauer E (2008) Specificity of the innate immune system and diversity of C-type lectin domain (CTLD) proteins in the nematode Caenorhabditis elegans. Immunobiology 213: 237–250. 18406370 10.1016/j.imbio.2007.12.004

[66] Pasche B, Kalaydjiev S, Franz TJ, Kremmer E, Gailus-Durner V, et al (2005) Sex-dependent susceptibility to Listeria monocytogenes infection is mediated by differential interleukin-10 production. Infect Immun 73: 5952–5960. 16113316 10.1128/IAI.73.9.5952-5960.2005 PMC1231091

[67] Chu DS, Liu H, Nix P, Wu TF, Ralston EJ, et al (2006) Sperm chromatin proteomics identifies evolutionarily conserved fertility factors. Nature 443: 101–105. 16943775 10.1038/nature05050 PMC2731558

[68] Pickett CL, Breen KT, Ayer DE (2007) A C. elegans Myc-like network cooperates with semaphorin and Wnt signaling pathways to control cell migration. Dev Biol 310: 226–239. 17826759 10.1016/j.ydbio.2007.07.034 PMC2077855

[69] Brenner S (1974) The genetics of Caenorhabditis elegans. Genetics 77: 71–94. 4366476 10.1093/genetics/77.1.71 PMC1213120

[70] Azevedo RB, Keightley PD, Lauren-Maatta C, Vassilieva LL, Lynch M, et al (2002) Spontaneous mutational variation for body size in Caenorhabditis elegans. Genetics 162: 755–765. 12399386 10.1093/genetics/162.2.755 PMC1462287

[71] Salomon MP, Ostrow D, Phillips N, Blanton D, Bour W, et al.. (2009) Comparing mutational and standing genetic variability for fitness and size in Caenorhabditis briggsae and C. elegans. Genetics 183: 685–692, 681SI–619SI. 10.1534/genetics.109.107383 PMC2766327 19667133

[72] Bolstad BM, Irizarry RA, Astrand M, Speed TP (2003) A comparison of normalization methods for high density oligonucleotide array data based on variance and bias. Bioinformatics 19: 185–193. 12538238 10.1093/bioinformatics/19.2.185

