# Supplementary material for: Sex Differences in Carbohydrate Metabolism Are Linked to Gene Expression in Caenorhabditis elegans
Source: PLoS One. 2012 Sep 11;7(9):e44748. doi: 10.1371/journal.pone.0044748 (PMC3439400; doi:10.1371/journal.pone.0044748)
Supplement: Table S1 — Body composition of males and hermaphrodites at different developmental stages in different male mutants. (DOC) [file pone.0044748.s001.doc]

Table S1. Body composition of males and hermaphrodites at different developmental stages in different male mutants^a^.

| Mutant | Stage | Sex | Protein [ng/worm] | Triglyceride [ng/worm] | Triglyceride to protein ratio |
| --- | --- | --- | --- | --- | --- |
| *him-8 GFP* | Young adult (66 h) | Hermaphrodite | 58.1 (± 4.7) | 12.3 (± 3.8) | 0.21 (± 0.07) |
|  |  | Male | 44.9 (± 4.6) | 12.8 (± 3.2) | 0.28 (± 0.08) |
|  |  | Male (adjusted) | 59.5 (± 3.3) | 17.2 (± 4.6) |  |
|  | Adult (76 h) | Hermaphrodite | 141.6 (± 13.8) | 16.4 (± 6.2) | 0.12 (± 0.05) |
|  |  | Male | 70.1 (± 9.9) | 7.9 (± 5.8) | 0.12 (± 0.10) |
|  |  | Male (adjusted) | 135.0 (± 18.1) | 15.9 (± 13.1) |  |
|  | One day adult (90 h) | Hermaphrodite | 194.1 (± 13.4) | 27.3 (± 1.9) | 0.14 (± 0.01) |
|  |  | Male | 75.3 (± 5.9) | 10.9 (± 0.5) | 0.14 (± 0.006) |
|  |  | Male (adjusted) | 162.7 (± 17.3) | 23.5 (± 1.5) |  |
|  |  |  |  |  |  |
| *fog-2* | One day adult (90 h) | Female | 211.3 (± 31.9) | 25.0 (± 7.7) | 0.12 (± 0.02) |
|  |  | Male | 92.7 (± 8.3) | 15.9 (± 4.5) | 0.17 (± 0.05) |
|  |  | Male (adjusted) | 200.2 (± 22.1) | 34.5 (± 10.4) |  |
|  |  |  |  |  |  |
| *him-8* | One day adult (90 h) | Hermaphrodite | 203.7 (± 15.0) | 23.4 (± 3.4) | 0.11 (± 0.009) |
|  |  | Male | 88.3 (± 3.1) | 9.8 (± 3.9) | 0.11 (± 0.05) |
|  |  | Male (adjusted) | 184.8 (± 7.4) | 20.4 (± 8.2) |  |
|  |  |  |  |  |  |
| *him-5* | One day adult (90 h) | Hermaphrodite | 156.9 (± 32.1) | 18.6 (± 4.0) | 0.12 (± 0.01) |
|  |  | Male | 69.5 (± 8.1) | 13.0 (± 8.4) | 0.18 (± 0.12) |
|  |  | Male (adjusted) | 135.3 (± 18.0) | 25.8 (± 17.3) |  |
|  |  |  |  |  |  |
| N2 | One day adult (90 h) | Hermaphrodite | 249.7 (± 9.9) | 31.3 (± 3.5) | 0.13 (± 0.02) |

^a^ Data is presented as mean (± SD) from 3-8 experiments. Male body composition parameters were adjusted to body volume so that they were comparable with hermaphrodite.
