# Supplementary material for: Sex Differences in Carbohydrate Metabolism Are Linked to Gene Expression in Caenorhabditis elegans
Source: PLoS One. 2012 Sep 11;7(9):e44748. doi: 10.1371/journal.pone.0044748 (PMC3439400; doi:10.1371/journal.pone.0044748)
Supplement: Table S2 — Glucose and trehalose content of males and hermaphrodites at different developmental stages in different male mutants. (DOC) [file pone.0044748.s002.doc]

Table S2. Glucose and trehalose content of males and hermaphrodites at different developmental stages in different male mutants^a^.

| Mutant | Stage | Sex | Trehalose  [ng/worm] | Glucose  [ng/worm] | Trehalose to glucose ratio |
| --- | --- | --- | --- | --- | --- |
| *him-8 GFP* | Young adult (66 h) | Hermaphrodite | 0.43 (± 0.17) | 1.87 (± 0.75) | 0.25 (± 0.09) |
|  |  | Male | 0.49 (± 0.18) | 1.50 (± 0.56) | 0.35 (± 0.08) |
|  |  | Male (adjusted) | 0.67 (± 0.30) | 2.03 (± 0.82) |  |
|  | Adult (76 h) | Hermaphrodite | 1.88 (± 1.09) | 5.74 (± 1.30) | 0.32 (± 0.16) |
|  |  | Male | 1.91 (± 0.66) | 1.64 (± 0.57) | 1.28 (± 0.58) |
|  |  | Male (adjusted) | 3.72 (± 1.35) | 3.21 (± 1.31) |  |
|  | One day adult (90 h) | Hermaphrodite | 2.74 (± 0.45) | 10.7 (± 1.39) | 0.26 (± 0.05) |
|  |  | Male | 3.61 (± 0.21) | 2.59 (± 0.24) | 1.40 (± 0.11) |
|  |  | Male (adjusted) | 7.78 (± 0.27) | 5.58 (± 0.34) |  |
|  |  |  |  |  |  |
| *fog-2* | One day adult (90 h) | Female | 2.84 (± 1.44) | 9.45 (± 2.21) | 0.29 (± 0.10) |
|  |  | Male | 3.26 (± 0.88) | 3.37 (± 0.10) | 0.97 (± 0.28) |
|  |  | Male (adjusted) | 7.08 (± 2.08) | 7.27 (± 0.11) |  |
|  |  |  |  |  |  |
| *him-8* | One day adult (90 h) | Hermaphrodite | 3.66 (± 1.83) | 8.86 (± 1.93) | 0.39 (± 0.15) |
|  |  | Male | 1.98 (± 0.92) | 2.50 (± 0.34) | 0.81 (± 0.46) |
|  |  | Male (adjusted) | 4.18 (± 2.09) | 5.22 (± 0.66) |  |
|  |  |  |  |  |  |
| *him-5* | One day adult (90 h) | Hermaphrodite | 2.83 (± 1.57) | 6.17 (± 2.17) | 0.43 (± 0.14) |
|  |  | Male | 1.57 (± 1.06) | 1.62 (± 1.11) | 1.27 (± 0.74) |
|  |  | Male (adjusted) | 3.06 (± 2.04) | 3.20 (± 2.23) |  |
|  |  |  |  |  |  |
| N2 | One day adult (90 h) | Hermaphrodite | 4.23 (± 0.46) | 12.5 (± 1.60) | 0.34 (± 0.03) |

^a^ Data is presented as mean (± SD) for 3-8 experiments. Male trehalose and glucose level were adjusted to body volume so that they were comparable to hermaphrodite.
