# Supplementary material for: Sex Differences in Carbohydrate Metabolism Are Linked to Gene Expression in Caenorhabditis elegans
Source: PLoS One. 2012 Sep 11;7(9):e44748. doi: 10.1371/journal.pone.0044748 (PMC3439400; doi:10.1371/journal.pone.0044748)
Supplement: Table S3 — The entire list of male-specific genes. (DOC) [file pone.0044748.s003.doc]

Table S3. The entire list of male-specific genes^a^.

|  |  | Male to hermaphrodite ratio | |  |
| --- | --- | --- | --- | --- |
| Gene ID | Gene name | Young adult | Adult | Description |
| B0280.16 | *sls-2.18* | 16.1 | 22.9 | transpliced leader sequence |
| C04B4.3 | *lips-2* | 204.0 | 638.3 | triacylglycerol lipase |
| C06E1.6 | *fipr-16* | 307.8 | 793.0 | fungus-induced protein |
| C07G3.10 |  | 85.3 | 114.3 | lipid binding (molecular function) |
| C10G8.2 |  | 79.5 | 144.8 | serine-type endopeptidase inhibitor activity |
| C14E2.4 |  | 81.6 | 313.5 | oxidoreductase activity (predicted) |
| C16A11.8 | *clec-135* | 582.3 | 1292.7 | c-type lectin, function unknown |
| C16E9.4b | *inx-1* | 18.4 | 21.1 | predicted member of the innexin family |
| C18F10.4 | *srg-1* | 14.4 | 16.2 | receptor-like protein, transmembrane receptor activity (predicted) |
| C28H8.7 |  | 28.5 | 83.7 | predicted secreted small molecules methylase |
| C29F9.5 |  | 37.3 | 54.8 | CREB binding protein/P300 and related TAZ Zn-finger proteins, histone acetyltransferase activity (predicted) |
| C32C4.5a | *mab-23* | 25.8 | 27.5 | transcription factor of the DM (DOUBLESEX/MAB-3) class |
| C32C4.5b | *mab-23* | 22.7 | 43.5 | transcription factor of the DM (DOUBLESEX/MAB-3) class |
| C32E8.6a |  | 76.1 | 152.2 | acyl-CoA synthetase |
| C34C6.8 | *ceh-7* | 10.8 | 11.2 | a homeodomain transcription factor |
| C35C5.2 |  | 12.1 | 14.3 | peptidase activity (predicted) |
| C37H5.11 | *cwp-2* | 54.5 | 339.3 | coexpressed with polycystins, encodes a nematode-specific protein that is coexpressed with lov-1 and pkd-2 |
| C37H5.4 | *cwp-3* | 44.6 | 110.4 | coexpressed with polycystins, encodes an unfamiliar protein predicted to be secreted and alleged to be coexpressed with polycystins |
| C39E9.6 | *scl-8* | 538.6 | 1034.2 | encodes a predicted extracellular protein that is a member of the C. elegans family of SCP/TAPS domain-containing proteins, defense-related protein containing SCP domain |
| C41G6.10 | *sri-25* | 10.5 | 41.3 | predicted olfactory G-protein coupled receptor |
| C42D4.11 | *clec-179* | 23.0 | 52.9 | binding |
| C45G9.10b |  | 103.1 | 475.3 | positive regulation of growth rate (phenotype) |
| C47A10.9 | *srh-287* | 13.1 | 13.0 | serpentine receptor, class H |
| C47G2.6 |  | 24.3 | 20.6 | Pseudogene |
| C50E3.15 |  | 11.1 | 15.1 | locomotion (phenotype) |
| C50E3.3 | *clec-213* | 34.1 | 44.6 | c-type lectin, function unknown |
| E03H12.3 | *clec-176* | 12.2 | 10.8 | binding |
| EEED8.11 | *clec-141* | 185.7 | 541.9 | binding |
| F02D10.1 | *col-183* | 77.0 | 264.2 | collagens (type IV and type XIII) and related proteins, structural constituent of cuticle |
| F02H6.6 |  | 44.3 | 66.6 | zinc metalloprotein |
| F07D10.1.1 | *rpl-11.2* | 144.4 | 1826.8 | encodes a large ribosomal subunit L11 protein; is predicted to function in protein biosynthesis |
| F07D10.1.2 | *rpl-11.2* | 142.3 | 1349.1 | encodes a large ribosomal subunit L11 protein; is predicted to function in protein biosynthesis |
| F07G6.1 | *dgn-3* | 39.6 | 55.7 | dystroglycan |
| F10A3.11 |  | 28.4 | 32.2 | lipid storage (phenotype) |
| F11D11.14 | *clec-250* | 19.8 | 15.4 | c-type lectin, binding (molecular function) |
| F11D11.5 | *clec-254* | 42.9 | 21.1 | c-type lectin, binding (molecular function) |
| F13E9.10 |  | 258.2 | 698.9 | predicted alpha-helical protein |
| F13E9.4 |  | 112.8 | 368.3 | predicted alpha-helical protein |
| F16G10.6 |  | 91.0 | 11.6 | extracellular protein with cysteine rich structures |
| F17A2.1 | *xtr-2* | 14.3 | 26.4 | encodes a protein with similarity to TRA-2A and TRA-2B in a region known as the MX region, hypothesized to be a protein-protein interaction domain involved in negatively regulating tra-2 activity in the germ line |
| F17B5.3 | *clec-109* | 132.6 | 1046.5 | c-type lectin, binding (molecular function) |
| F17B5.5 | *clec-110* | 186.2 | 816.1 | c-type lectin, function unknown |
| F22B7.2 | *flp-23* | 109.4 | 959.2 | FMRF-like peptide |
| F26A1.12 | *clec-157* | 356.9 | 201.2 | c-type lectin, binding (molecular function) |
| F26D11.9 | *clec-217* | 316.0 | 1448.0 | c-type lectin, function unknown |
| F26D2.7 | *str-55* | 87.2 | 46.2 | 7-transmembrane olfactory receptor |
| F26F2.6 | *clec-263* | 320.2 | 999.5 | c-type lectin, function unknown |
| F28B1.1 | *hpo-33* | 159.8 | 1352.6 | hypersensitive to pore-forming toxin |
| F32B4.6 |  | 746.7 | 1450.9 | predicted alpha/beta hydrolase, acting on ester bonds |
| F33E2.3 | *clec-102* | 20.6 | 62.8 | c-type lectin, binding (molecular function) |
| F33E2.6 |  | 42.9 | 157.8 | collagens (type IV and type XIII) and related proteins |
| F33H1.5 | *srd-1* | 15.7 | 14.0 | encodes a seven transmembrane chemosensory receptor |
| F35C5.3 |  | 10.9 | 397.4 | reproduction (phenotype) |
| F35D11.7 | *clec-136* | 139.4 | 1309.1 | c-type lectin, function unknown |
| F36G9.11 | *clec-232* | 252.3 | 633.3 | c-type lectin, binding (molecular function) |
| F40H6.5 |  | 21.8 | 22.7 | sugar binding |
| F43C11.1 |  | 311.6 | 1759.9 | extracellular protein with cysteine rich structures |
| F43C11.12 |  | 179.8 | 1512.7 | extracellular protein with cysteine rich structures |
| F43C11.2 |  | 134.9 | 924.5 | extracellular protein with cysteine rich structures |
| F45C12.9 |  | 87.6 | 994.7 | extracellular protein with cysteine rich structures |
| F45G2.6 | *trf-1* | 28.1 | 40.9 | encodes a protein with a meprin-associated Traf homology (MATH) domain that may be involved in apoptosis |
| F46A8.3 |  | 312.0 | 855.3 | Galectin, galactose-binding lectin, sugar binding |
| F46A8.5 |  | 337.1 | 1335.7 | Galectin, galactose-binding lectin, sugar binding |
| F46A8.8 |  | 366.4 | 1046.8 | sugar binding, Galectin |
| F47G4.1 | *clec-113* | 13.9 | 12.0 | c-type lectin, function unknown |
| F49C5.10 |  | 74.6 | 379.7 | involved in purine ribonucleoside monophosphate biosynthetic process, deaminase activity |
| F49F1.10 |  | 251.4 | 1195.6 | Galectin, galactose-binding lectin, sugar binding |
| F49F1.11 |  | 323.8 | 1330.1 | Galectin, galactose-binding lectin, sugar binding |
| F49F1.9 |  | 460.5 | 884.5 | Galectin, galactose-binding lectin, sugar binding |
| F49H6.10 | *srz-93* | 13.4 | 16.1 | Serpentine Receptor, class Z, pseudogene |
| F56H1.2 | *nhr-266* | 16.4 | 13.9 | nuclear hormone receptor family,transcription factor activity |
| F57E7.5 |  | 10.1 | 32.1 | pseudogene |
| F58A4.5 | *clec-161* | 267.2 | 952.3 | c-type lectin, binding (molecular function) |
| F58F9.6 |  | 538.5 | 1390.4 | zinc metalloprotein |
| H25K10.3 | *srv-11* | 10.9 | 17.4 | serpentine receptor, class V, transmembrane receptor activity |
| K02E7.t1 |  | 62.1 | 121.0 | transfer-RNA |
| K03B8.2 | *nas-17* | 598.6 | 1335.3 | encodes an astacin-like metalloprotease |
| K03B8.5 | *nas-19* | 337.8 | 1556.8 | encodes an astacin-like metalloprotease |
| K04H8.1 | *clec-116* | 28.0 | 33.3 | binding |
| K07B1.1 | *try-5* | 41.5 | 76.5 | trypsin-like protease |
| K07C6.4 | *cyp-35B1* | 28.0 | 12.9 | cytochrome P450 family, oxidoreductase activity |
| M162.1 | *clec-259* | 148.9 | 238.4 | binding |
| M7.10 |  | 391.6 | 1062.1 | predicted alpha-helical protein |
| R03H10.4 |  | 133.9 | 1162.3 | extracellular protein with cysteine rich structures, lipid storage (phenotype) |
| R03H10.5 |  | 19.6 | 19.0 | extracellular protein with cysteine rich structures |
| R05A10.5 |  | 15.1 | 48.5 | zinc metalloprotein |
| R05H10.7 |  | 413.9 | 425.8 | metallocarboxypeptidase activity |
| R11E3.4 | *set-15* | 80.0 | 202.0 | encodes a SET domain-containing protein required for normally short lifespan; Histone H3 (Lys9) methyltransferase SUV39H1/Clr4, required for transcriptional silencing, protein binding |
| R13F6.2 | *clec-159* | 145.5 | 198.7 | c-type lectin, unknown function |
| R13F6.8 | *clec-158* | 189.4 | 925.3 | c-type lectin, unknown function |
| T05H4.8 |  | 115.9 | 1610.0 | pseudogene |
| T12A2.5 |  | 511.9 | 2092.9 | lipid storage (moleculare function) |
| T12A7.3 | *scl-18* | 159.7 | 1323.1 | CAP domain, cysteine-rich secretory protein family |
| T19B10.12 |  | 470.8 | 1744.2 | TonB box, conserved site (protein domain) |
| T20D3.1 | *clec-183* | 247.9 | 578.6 | c-type lectin, binding |
| T21C9.11 |  | 11.3 | 10.8 | lipid storage (phenotype) |
| T22G5.1 |  | 500.5 | 1274.2 | glycerophosphoryl diester phosphodiesterase activity |
| T23F2.3 |  | 140.7 | 989.4 | Stress responsive protein |
| T23G4.5 |  | 165.8 | 1137.9 | predicted alpha-helical protein |
| T24D8.3 | *nlp-22* | 32.5 | 60.4 | neuropeptide-like protein |
| T28A8.2 |  | 24.4 | 48.5 | predicted galactoside 2-alpha-L-fucosyltransferase activity |
| W02B3.5 |  | 65.1 | 381.6 | fukutin-related (protein domain) |
| W02B3.7 |  | 85.8 | 122.0 | fukutin-related (protein domain) |
| W02D7.12 | *clec-219* | 183.1 | 1163.3 | c-type lectin, unknown function |
| W04A4.3 |  | 109.1 | 166.4 | gamma-interferon inducible lysosomal thiol reductase |
| W09G10.5 | *clec-126* | 121.8 | 171.5 | c-type lectin, binding |
| W09G10.6 | *clec-125* | 176.6 | 404.7 | c-type lectin, binding |
| W10G11.11 | *clec-134* | 1593.0 | 1063.3 | c-type lectin, unknown function |
| W10G11.12 | *clec-133* | 220.9 | 1046.3 | c-type lectin, unknown function |
| W10G11.13 | *clec-132* | 140.9 | 167.2 | c-type lectin, binding |
| W10G11.14 | *clec-130* | 341.0 | 772.0 | c-type lectin, binding |
| W10G11.15 | *clec-129* | 496.1 | 1421.1 | c-type lectin, binding |
| Y102A5C.10 | *fbxa-204* | 63.0 | 91.0 | F-box A protein, pseudogene |
| Y116A8A.3 | *clec-193* | 57.3 | 65.2 | c-type lectin, unknown function |
| Y17D7B.6 | *clec-256* | 19.3 | 33.8 | c-type lectin, unknown function |
| Y17D7B.8 | *clec-257* | 33.8 | 51.9 | c-type lectin, unknown function |
| Y26D4A.4.1 | *clec-107* | 903.5 | 1582.3 | c-type lectin, unknown function |
| Y26D4A.4.2 | *clec-107* | 860.8 | 1138.6 | c-type lectin, unknown function |
| Y26D4A.6 | *clec-108* | 145.8 | 508.9 | c-type lectin, binding |
| Y38E10A.2 |  | 46.7 | 45.5 | unknown, lipid storage (phenotype) |
| Y39B6A.t4 |  | 140.2 | 79.4 | t-RNA |
| Y43F8B.19 |  | 84.2 | 261.0 | oxidoreductase activity |
| Y46D2A.3 |  | 86.6 | 325.2 | zinc metalloprotein |
| Y47D7A.10 | *hpo-37* | 462.4 | 606.1 | hypersensitive to pore-forming toxin |
| Y51A2D.1 |  | 90.5 | 398.5 | cysteine-type peptidase activity |
| Y52B11A.5 | *clec-92* | 279.5 | 935.9 | c-type lectin, binding |
| Y53C12B.5a | *mab-3* | 25.0 | 44.0 | encodes a DM (Doublesex and MAB-3) domain-containing protein, transcription factor activity |
| Y55F3C.5 | *clec-164* | 24.5 | 35.4 | c-type lectin, unknown function |
| Y59H11AR.5 | *clec-181* | 256.1 | 729.2 | c-type lectin, binding |
| Y71A12B.6 | *clec-112* | 14.3 | 17.4 | c-type lectin, unknown function |
| Y73F8A.1 | *pkd-2* | 41.2 | 120.3 | encodes an ortholog of human PKD2 (mutated in autosomal dominant polycystic kidney disease), calcium ion binding-ion channel activity |
| Y82E9BL.12 |  | 16.1 | 71.6 | predicted secreted small molecules methylase |
| ZC15.6 | *clec-261* | 232.0 | 1092.4 | c-type lectin, binding |
| ZC334.8 | *ins-25* | 17.8 | 18.7 | insulin related |
| ZK1025.9 | *nhr-113* | 16.6 | 72.5 | nuclear hormone receptor family, transcription factor activity |
| ZK1248.1 |  | 98.7 | 327.5 | metalloendopeptidase activity |
| ZK1290.1 |  | 425.6 | 1124.4 | lipid storage (phenotype) |
| ZK39.2 | *clec-95* | 220.8 | 925.5 | c-type lectin, binding |
| ZK39.4 | *clec-93* | 43.0 | 109.7 | c-type lectin, binding |
| ZK39.5 | *clec-96* | 257.5 | 898.3 | c-type lectin, binding |
| ZK945.9 | *lov-1* | 55.6 | 111.6 | encodes an ortholog of human PKD1 (mutated in autosomal dominant polycystic kidney disease) protein binding |
| B0207.5 |  | 144.4 | 537.4 | unknown |
| B0228.8 |  | 76.6 | 441.2 | unknown |
| C01G10.18 |  | 202.7 | 88.2 | unknown |
| C03B1.4 |  | 14.2 | 14.6 | unknown |
| C04B4.6 |  | 43.2 | 57.2 | unknown |
| C06A12.8 |  | 74.0 | 403.1 | unknown |
| C06E2.9 |  | 171.2 | 546.7 | unknown |
| C08E3.14 |  | 72.0 | 167.6 | unknown |
| C14C6.13 |  | 17.4 | 72.4 | unknown |
| C16C10.9 |  | 16.0 | 10.7 | unknown |
| C16C8.10 |  | 232.3 | 2100.0 | unknown |
| C16C8.17 |  | 157.1 | 1473.5 | unknown |
| C16C8.7 |  | 195.7 | 1175.4 | unknown |
| C16C8.8 |  | 201.8 | 1832.1 | unknown |
| C17H12.11.1 |  | 73.0 | 173.7 | unknown |
| C24A3.9 |  | 57.3 | 48.3 | unknown |
| C25F9.8 |  | 101.3 | 853.3 | unknown |
| C26E1.1 |  | 23.8 | 71.4 | unknown |
| C27C7.5 |  | 15.3 | 12.8 | unknown |
| C35E7.7 |  | 104.5 | 601.5 | unknown |
| C44B12.9 |  | 140.4 | 591.9 | unknown |
| C46E10.3 |  | 23.6 | 45.8 | unknown |
| C48B4.12a |  | 483.8 | 898.4 | unknown |
| C49C8.6 |  | 51.3 | 320.1 | unknown |
| C50H11.8 |  | 12.6 | 15.8 | unknown |
| C55C3.7 |  | 36.6 | 35.6 | unknown |
| D1022.2 |  | 141.0 | 921.2 | unknown |
| E02H9.1 |  | 129.8 | 374.2 | unknown |
| F07G6.8 |  | 192.2 | 1181.7 | unknown |
| F13A2.5 |  | 43.9 | 190.8 | unknown |
| F13E9.9 |  | 51.7 | 334.0 | unknown |
| F16G10.10 |  | 281.8 | 1703.5 | unknown |
| F16G10.2 |  | 51.3 | 442.3 | unknown |
| F17E9.15 |  | 129.3 | 480.4 | unknown |
| F18E9.7 |  | 445.6 | 1848.1 | unknown |
| F18G5.5 |  | 20.8 | 45.8 | unknown |
| F19H6.5 |  | 115.9 | 480.6 | unknown |
| F19H6.6 |  | 109.2 | 469.0 | unknown |
| F25B3.2 |  | 26.0 | 305.6 | unknown |
| F25D7.5 |  | 30.4 | 78.6 | unknown |
| F26C11.3b |  | 62.6 | 232.9 | unknown |
| F26C11.4 |  | 149.2 | 988.1 | unknown |
| F28B1.2 |  | 91.2 | 777.7 | unknown |
| F28B1.3 |  | 101.1 | 196.3 | unknown |
| F28B1.9 |  | 220.7 | 948.8 | unknown |
| F33D11.6 |  | 46.0 | 55.1 | unknown |
| F35C5.4 |  | 32.8 | 48.5 | unknown |
| F38E1.10 |  | 131.9 | 1528.1 | unknown |
| F38E11.11 |  | 127.1 | 694.1 | unknown |
| F40E12.1 |  | 19.7 | 16.0 | unknown |
| F40G9.7.1 |  | 174.4 | 1446.0 | unknown |
| F42A6.2 |  | 458.9 | 632.0 | unknown |
| F44A2.7 |  | 66.6 | 24.4 | unknown |
| F46B3.9 |  | 23.3 | 564.1 | unknown |
| F47C12.11 |  | 64.0 | 74.7 | unknown |
| F47C12.6 |  | 110.3 | 902.2 | unknown |
| F47C12.7 |  | 137.8 | 452.5 | unknown |
| F47G9.6 |  | 18.5 | 15.6 | unknown |
| F49C5.7 |  | 10.6 | 37.2 | unknown |
| F55G11.10 |  | 33.1 | 103.9 | unknown |
| F56C4.2 |  | 12.5 | 32.4 | unknown |
| F58F9.8 |  | 300.0 | 454.8 | unknown |
| F59A1.16 |  | 411.5 | 1451.2 | unknown |
| F59A1.6 |  | 125.3 | 1092.5 | unknown |
| F59A6.3 |  | 69.0 | 74.6 | unknown |
| H23L24.1 |  | 47.0 | 64.6 | unknown |
| K03B8.11 |  | 499.1 | 396.8 | unknown |
| K03B8.14 |  | 342.4 | 734.6 | unknown |
| K04F1.8 |  | 64.1 | 234.2 | unknown |
| K08C9.6 |  | 339.3 | 247.9 | unknown |
| K08C9.9 |  | 57.4 | 93.0 | unknown |
| K09C8.2 |  | 110.5 | 1395.8 | unknown |
| K12H6.5 |  | 803.7 | 2061.8 | unknown |
| K12H6.8 |  | 593.6 | 1154.1 | unknown |
| M02D8.7 |  | 19.5 | 36.4 | unknown |
| M04D8.5 |  | 14.2 | 19.3 | unknown |
| R04A9.1 |  | 11.7 | 13.0 | unknown |
| R09E10.8 |  | 123.2 | 1381.5 | unknown |
| R11G10.4 |  | 233.7 | 1192.2 | unknown |
| R13A1.1 |  | 13.2 | 111.0 | unknown |
| R160.6 |  | 20.1 | 17.0 | unknown |
| R53.8 |  | 161.0 | 948.1 | unknown |
| T02D1.7 |  | 534.6 | 1159.4 | unknown |
| T04A6.2 |  | 16.7 | 30.1 | unknown |
| T05A8.7 |  | 45.6 | 70.3 | unknown |
| T10D4.15 |  | 112.0 | 523.5 | unknown |
| T10D4.7 |  | 149.6 | 429.2 | unknown |
| T12A7.9 |  | 13.4 | 69.8 | unknown |
| T20F5.8 |  | 25.4 | 21.1 | unknown |
| T21E8.7 |  | 44.1 | 40.2 | unknown |
| T22C1.12 |  | 111.5 | 1030.9 | unknown |
| T24E12.12 |  | 17.2 | 55.9 | unknown |
| T26E3.6 |  | 235.2 | 1465.7 | unknown |
| T28B4.2 |  | 10.1 | 15.0 | unknown |
| W03G9.7 |  | 10.3 | 15.3 | unknown |
| W04G5.7 |  | 13.7 | 14.5 | unknown |
| W06D12.6 |  | 63.8 | 61.8 | unknown |
| W06G6.12 |  | 12.6 | 12.8 | unknown |
| W07G1.1 |  | 13.6 | 13.3 | unknown |
| W07G1.7 |  | 186.7 | 614.5 | unknown |
| W07G4.8 |  | 14.7 | 12.1 | unknown |
| Y105C5A.9b |  | 80.7 | 38.4 | unknown |
| Y110A2AL.6 |  | 129.7 | 1641.0 | unknown |
| Y116F11A.3 |  | 304.2 | 1404.9 | unknown |
| Y17G7B.23 |  | 50.4 | 81.9 | unknown |
| Y18D10A.2 |  | 132.9 | 853.3 | unknown |
| Y25C1A.2 |  | 145.3 | 526.1 | unknown |
| Y37F4.3 |  | 110.5 | 890.6 | unknown |
| Y38H6C.18 |  | 44.9 | 51.7 | unknown |
| Y41D4A.1 |  | 25.3 | 86.1 | unknown |
| Y43F8B.10 |  | 22.7 | 35.2 | unknown |
| Y43F8C.15 |  | 58.2 | 236.7 | unknown |
| Y43F8C.16 |  | 179.6 | 1412.1 | unknown |
| Y43F8C.23 |  | 77.2 | 266.9 | unknown |
| Y46G5A.23 |  | 333.2 | 1237.7 | unknown |
| Y47D7A.11 |  | 694.9 | 732.8 | unknown |
| Y48G8AR.3 |  | 48.6 | 105.5 | unknown |
| Y48G9A.6 |  | 59.9 | 165.5 | unknown |
| Y49F6B.13 |  | 228.8 | 1652.3 | unknown |
| Y49F6B.6 |  | 743.1 | 1546.7 | unknown |
| Y53G8AL.3 |  | 40.0 | 72.9 | unknown |
| Y59E1B.1 |  | 12.6 | 14.1 | unknown |
| Y62H9A.2 |  | 45.0 | 57.1 | unknown |
| Y62H9A.8 |  | 35.7 | 167.1 | unknown |
| Y64G10A.2 |  | 218.5 | 1293.1 | unknown |
| Y67A10A.11 |  | 46.8 | 146.1 | unknown |
| Y67A10A.2 |  | 29.3 | 36.1 | unknown |
| Y67D8C.7 |  | 244.6 | 121.5 | unknown |
| Y68A4A.13 |  | 25.9 | 113.2 | unknown |
| Y6G8.14 |  | 590.7 | 1130.4 | unknown |
| Y6G8.16 |  | 280.6 | 890.6 | unknown |
| Y71G12B.5 |  | 18.1 | 10.1 | unknown |
| Y73F8A.10 |  | 181.8 | 1589.1 | unknown |
| Y75B12B.13 |  | 242.2 | 689.0 | unknown |
| Y7A5A.11 |  | 23.3 | 31.0 | unknown |
| Y7A5A.9 |  | 14.8 | 17.1 | unknown |
| Y82E9BL.1 |  | 27.4 | 24.6 | unknown |
| Y82E9BR.7 |  | 57.2 | 46.1 | unknown |
| Y82E9BR.9 |  | 41.5 | 43.7 | unknown |
| ZC178.2 |  | 131.4 | 898.7 | unknown |
| ZC204.17 |  | 233.5 | 890.0 | unknown |
| ZC328.5 |  | 281.9 | 244.2 | unknown |
| ZK1290.11 |  | 723.9 | 985.9 | unknown |
| ZK39.9 |  | 901.8 | 1489.4 | unknown |

^a^ Information about genes was obtained from WormBase (WS229, www.wormbase.org).
