# Supplementary material for: Sex Differences in Carbohydrate Metabolism Are Linked to Gene Expression in Caenorhabditis elegans
Source: PLoS One. 2012 Sep 11;7(9):e44748. doi: 10.1371/journal.pone.0044748 (PMC3439400; doi:10.1371/journal.pone.0044748)
Supplement: Table S4 — The entire list of hermaphrodite-specific genes. (DOC) [file pone.0044748.s004.doc]

Table S4. The entire list of hermaphrodite-specific genes^a^.

|  |  | Hermaphrodite to male ratio | |  |
| --- | --- | --- | --- | --- |
| Gene ID | Gene name | Young adult | Adult | Description |
| C01C10.3 | *acl-12* | 11.5 | 28.2 | acyltransferase activity |
| C03E10.5 | *clec-223* | 494.6 | 177.8 | c-type lectin, binding(molecular function) |
| C18D11.7 |  | 56.4 | 13.4 | protein binding (molecular function) |
| C27C12.3 |  | 25.2 | 80.3 | encodes a novel protein that is conserved in C. elegans; is expressed in the proximal germline |
| C29G2.1 |  | 42.6 | 161.5 | C2H2-type Zn-finger |
| C30F2.2 |  | 26.0 | 12.4 | similar to ARD GTP-binding proteins |
| C32B5.10 | *fbxc-32* | 84.1 | 191.9 | F-box C protein |
| C32B5.6 |  | 23.2 | 71.7 | expression is seen only in adults in two symmetrical neurons, whose cell bodies are located on the ventral side of the terminal bulb |
| C36C9.1 |  | 16.4 | 65.1 | expression is seen in pairs of nuclei around the isthmus of the pharynx, nuclei are probably neuronal, in older larvae and adults weak expression is seen in the pharynx |
| C44C3.8 | *srh-85* | 152.3 | 197.9 | serpentine receptor, class H, pseudogene |
| C46E10.8 |  | 21.5 | 69.5 | zinc ion binding |
| C46E10.9 |  | 54.0 | 118.0 | expressed in unidentified cells running parallel to the pharynx, zinc ion binding |
| C47F8.1 |  | 24.4 | 211.1 | encodes a protein containing an F-box, a motif predicted to mediate protein-protein interactions either with homologs of yeast Skp-1p or with other proteins, protein binding (molecular function) |
| C50E3.11 |  | 57.8 | 144.3 | folic acid binding, reduced folate carrier activity |
| C50E3.12 |  | 173.1 | 179.2 | locomotion (phenotype) |
| C53B7.7 |  | 120.0 | 80.1 | encodes a neprilysin, neprilysins are thermolysin-like zinc metallopeptidases |
| E01G6.3 |  | 54.7 | 42.1 | hydrolase activity |
| E02H4.6 |  | 120.3 | 162.0 | protein kinase activity, protein serine/threonine kinase activity |
| F08A8.3 |  | 33.2 | 44.6 | acyl-CoA dehydrogenase activity |
| F08F3.6 |  | 110.9 | 163.2 | encodes a protein containing an F-box, a motif predicted to mediate protein-protein interactions either with homologs of yeast Skp-1p or with other proteins |
| F09A5.9 | *ttr-34* | 140.3 | 67.0 | transthyretin-related family domain |
| F14B8.3 | *pes-23* | 97.2 | 55.6 | patterned expression site |
| F14D7.2 |  | 138.9 | 146.7 | morphogenesis of an epithelium (biologic function |
| F15A2.5 | *efn-3* | 33.8 | 31.1 | Eph(f)rin, encodes a potential GPI-modified ephrin that is, with EFN-2, is required for normal epidermal organization in the male tail |
| F15E6.6 |  | 239.3 | 65.3 | oxidoreductase activity |
| F16H11.3 |  | 259.3 | 244.1 | nucleoside transmembrane transporter activity |
| F17A9.6 | *ceh-49* | 77.0 | 166.0 | C. elegans homeobox, encodes a divergent ONECUT class CUT homeobox protein with a single N-terminal cut domain; specific DNA binding-transcription factor activity |
| F22G12.t1 |  | 34.5 | 35.6 | t-RNA |
| F25H5.9 |  | 85.7 | 72.7 | only transcript, no protein |
| F29G9.7 |  | 21.8 | 50.8 | encodes a protein containing an F-box, a motif predicted to mediate protein-protein interactions either with homologs of yeast Skp-1p or with other proteins |
| F31B12.3f | *frm-9* | 52.5 | 42.0 | FERM domain (protein4.1-ezrin-radixin-moesin) family |
| F31F6.1 |  | 42.3 | 29.0 | encodes a novel protein that is conserved in C. elegans |
| F31F6.2 |  | 26.6 | 103.8 | encodes a novel protein that is conserved in C. elegans, loss of function via large-scale RNAi results in increased fat content |
| F35C5.2 | *sra-14* | 10.1 | 16.5 | serpentine receptor, class A, transmembrane receptor activity |
| F39F10.3 |  | 24.2 | 82.8 | protein kinase activity |
| F45C12.15.1 | *ceh-83* | 17.1 | 42.6 | C. elegans homeobox, transcription factor activity |
| F45C12.15.3 | *ceh-83* | 14.0 | 42.5 | C. elegans homeobox, transcription factor activity |
| F45C12.8 | *fbxa-184* | 11.7 | 40.4 | F-box A protein |
| F45D11.9 | *fbxc-42* | 29.7 | 151.9 | F-box C protein |
| F47A4.3a | *rrc-1* | 58.2 | 10.6 | RhoGAP for Rac-1 and Cdc-42, is expressed in all life stages with high expression in L1 to L4 larvae stages |
| F47A4.3c | *rrc-1* | 63.9 | 11.0 | RhoGAP for Rac-1 and Cdc-42, is expressed in all life stages with high expression in L1 to L4 larvae stages |
| F47H4.1 | *lsy-27* | 114.5 | 189.6 | laterally symmetric (defective in lateral asymmetry)), encodes a nematode specific, fast evolving family of C2H2 zinc finger transcription factor |
| F49B2.2 | *fbxb-67* | 12.9 | 37.3 | F-box B protein |
| F52D2.2 | *rgs-8.1* | 17.9 | 78.2 | regulator of G protein signaling, signal transducer activity |
| F54D10.7 |  | 245.3 | 123.1 | protein binding, ubiquitin family protein domain |
| F55B11.5 |  | 1406.2 | 181.9 | determination of adult life span (phenotype) |
| K02B9.1 | *meg-1* | 311.7 | 159.3 | maternal effect germ-cell defective, encodes a novel protein that localizes exclusively to P granules; is required for germline development and normal levels of fertility |
| K04C1.5 |  | 216.0 | 235.9 | protein kinase activity, protein serine/threonine kinase activity |
| K07A1.6 |  | 769.1 | 178.8 | encodes a putative secreted TIL-domain protease inhibitor paralogous to SWM-1, ISL-1, and the products of 11 other C. elegans genes |
| M01G12.8 |  | 14.8 | 114.4 | pseudogene |
| M199.4 | *clec-190* | 245.0 | 192.8 | c-type lectin, unknown function |
| M199.5 | *col-135* | 779.4 | 176.0 | collagen, collagen triple helix repeat protein domain |
| R02C2.2 | *kin-34* | 11.6 | 219.6 | protein kinase activity-protein serine/threonine kinase activity |
| R03G8.6 |  | 382.9 | 177.9 | metallopeptidase activity and zinc ion binding |
| R07C3.9 | *fbxc-31* | 12.8 | 57.4 | F-box C protein |
| T01E8.2 | *ref-1* | 16.5 | 28.0 | regulator of fusion, encodes a protein with two basic helix-loop-helix (bHLH) domains that is distantly related to the hairy/Enhancer of split subfamily of bHLH transcription factors, regulation of transcription |
| T02G6.5 |  | 135.6 | 151.6 | encodes a protein containing an F-box, a motif predicted to mediate protein-protein interactions either with homologs of yeast Skp-1p or with other proteins |
| T04D3.1 |  | 45.4 | 125.2 | uncharacterized coiled-coil containing protein |
| T05A10.3 | *ttr-14* | 514.1 | 144.2 | transthyretin-related family domain, cell migration, gonad development, receptor-mediated endocytosis (phenotype) |
| T05E7.1 |  | 96.5 | 83.6 | peroxisomal long chain acyl-CoA thioesterase I/predicted bile acid-CoA-amino acid N-acyltransferase |
| T05G11.1 | *pzf-1* | 206.4 | 127.8 | paired zinc finger protein, encodes a protein with three pairs of C2H2 zinc fingers |
| T07C4.6 | *tbx-9* | 37.6 | 94.7 | T box family, RNA polymerase II transcription factor activity |
| T10C6.10a |  | 29.3 | 133.5 | encodes a protein containing an F-box, a motif predicted to mediate protein-protein interactions either with homologs of yeast Skp-1p or with other proteins, lipid storage (phenotype) |
| T10C6.10b |  | 157.3 | 156.9 | encodes a protein containing an F-box, a motif predicted to mediate protein-protein interactions either with homologs of yeast Skp-1p or with other proteins, lipid storage (phenotype) |
| T10C6.7 |  | 10.1 | 29.7 | encodes a protein containing an F-box, a motif predicted to mediate protein-protein interactions either with homologs of yeast Skp-1p or with other proteins |
| T13B5.9 |  | 56.4 | 56.5 | metalloendopeptidase activity and zinc ion binding |
| T28H11.4 | *pes-1* | 10.1 | 34.5 | patterned expression site, transcription factor activity |
| W02D9.5 | *ssp-37* | 1013.1 | 170.9 | sperm specific family, class P, contains major sperm protein (MSP) domain, structural molecule activity |
| Y105E8B.10 |  | 34.2 | 44.2 | pseudogene |
| Y116A8C.22 |  | 14.0 | 133.1 | AT hook-like protein domain, DNA, protein and zinc ion binding |
| Y11D7A.13 | *flh-3* | 35.8 | 97.2 | FLYWCH zinc finger transcription factor homolog, transcription factor activity |
| Y27F2A.3a | *sri-40* | 30.1 | 102.0 | serpentine receptor, class I, predicted olfactory G-protein coupled receptor |
| Y27F2A.3b | *sri-40* | 28.4 | 97.5 | serpentine receptor, class I, predicted olfactory G-protein coupled receptor |
| Y54G11A.16 | *srh-43* | 483.6 | 104.1 | serpentine receptor, class H, pseudogene |
| Y6G8.3 | *ztf-25* | 257.4 | 239.9 | zinc finger putative transcription factor family, Ubiquitous strong expression is seen in early and comma stage embryos |
| ZC53.7 | *rgs-9* | 170.7 | 192.2 | regulator of G protein signaling, signal transducer activity |
| ZK1127.1 | *nos-2* | 183.8 | 161.5 | nanos related, encodes one of three genes in C. elegans that contains a putative zinc-binding domain similar to the one found in Drosophila nanos |
| ZK546.15 | *try-1* | 93.6 | 120.9 | trypsin-like protease), serine-type endopeptidase activity |
| ZK637.11 | *cdc-25.3* | 527.2 | 173.9 | cell division cycle related, encodes a tyrosine phosphatase that is a member of the cell division cycle 25 (CDC25) family of cell cycle regulators |
| ZK829.5 | *tbx-36* | 91.1 | 98.8 | T box family, transcription factor activity |
| B0281.4 |  | 36.5 | 129.0 | unknown |
| B0545.4 |  | 70.0 | 73.0 | unknown |
| C08A9.10 |  | 1754.5 | 191.3 | unknown |
| C09G9.8b |  | 14.6 | 22.2 | unknown |
| C10A4.4 |  | 17.7 | 10.8 | unknown |
| C14F11.4a |  | 333.8 | 71.5 | unknown |
| C14F11.4b |  | 317.0 | 73.5 | unknown |
| C17E7.12 |  | 345.6 | 171.7 | unknown |
| C17E7.4 |  | 391.8 | 184.8 | unknown |
| C17E7.9b |  | 128.6 | 144.2 | unknown |
| C17G1.2 |  | 340.3 | 173.8 | unknown |
| C30G7.4.2 |  | 50.4 | 42.5 | unknown |
| C45H4.14a |  | 32.8 | 172.5 | unknown |
| C46C2.5 |  | 79.0 | 58.0 | unknown |
| C50E3.13 |  | 138.1 | 193.2 | unknown |
| D1086.10a |  | 1392.1 | 160.7 | unknown |
| D1086.10c |  | 993.2 | 231.7 | unknown |
| D1086.11a |  | 145.9 | 96.3 | unknown |
| D1086.7 |  | 1592.1 | 188.9 | unknown |
| E02H9.7 |  | 356.4 | 164.9 | unknown |
| F02E8.4 |  | 207.1 | 170.8 | unknown |
| F02H6.1 |  | 10.2 | 46.8 | unknown |
| F02H6.2 |  | 133.7 | 219.3 | unknown |
| F02H6.3a.1 |  | 109.9 | 174.0 | unknown |
| F07G6.10 |  | 97.0 | 38.6 | unknown |
| F14H3.3 |  | 90.6 | 176.9 | unknown |
| F22E5.17 |  | 76.4 | 90.5 | unknown |
| F22F4.5 |  | 238.2 | 200.7 | unknown |
| F25H5.8 |  | 31.4 | 13.6 | unknown |
| F26G5.1b |  | 177.2 | 194.9 | unknown |
| F30A10.13b.2 |  | 128.2 | 94.5 | unknown |
| F36A4.5 |  | 277.3 | 88.9 | unknown |
| F39G3.2 |  | 27.0 | 14.5 | unknown |
| F40G12.11 |  | 226.2 | 157.2 | unknown |
| F52D1.2 |  | 36.9 | 148.6 | unknown |
| F54D11.3 |  | 33.8 | 59.5 | unknown |
| F54D5.5a |  | 23.1 | 81.9 | unknown |
| F54D5.5b |  | 22.0 | 80.8 | unknown |
| F54F7.9 |  | 17.8 | 13.9 | unknown |
| F58E1.13 |  | 17.5 | 47.8 | unknown |
| H11L12.1 |  | 11.1 | 19.0 | unknown |
| K01G12.3 |  | 21.5 | 99.7 | unknown |
| K08H2.3 |  | 29.7 | 75.3 | unknown |
| K09A9.8 |  | 18.5 | 17.9 | unknown |
| K09D9.12 |  | 45.8 | 102.3 | unknown |
| K09E3.7 |  | 12.5 | 74.8 | unknown |
| M01G12.14 |  | 74.1 | 26.2 | unknown |
| R04D3.3.1 |  | 44.6 | 126.6 | unknown |
| R04D3.3.2 |  | 58.7 | 141.2 | unknown |
| R04D3.4 |  | 165.7 | 188.1 | unknown |
| R05G9.3 |  | 77.3 | 213.6 | unknown |
| R09A8.1a |  | 55.1 | 249.6 | unknown |
| R09A8.1b |  | 22.9 | 74.9 | unknown |
| R09F10.8.1 |  | 34.5 | 107.2 | unknown |
| R09F10.8.2 |  | 35.2 | 112.7 | unknown |
| R10E4.6 |  | 25.5 | 39.9 | unknown |
| T06D4.1a |  | 283.9 | 167.1 | unknown |
| T06D4.1b.3 |  | 450.8 | 226.5 | unknown |
| T11F8.2.1 |  | 112.1 | 101.3 | unknown |
| T12B5.14 |  | 928.3 | 272.2 | unknown |
| T12B5.15 |  | 2541.7 | 193.3 | unknown |
| T16G12.8 |  | 14.2 | 43.3 | unknown |
| T19H5.7 |  | 72.7 | 111.7 | unknown |
| W04E12.2 |  | 22.5 | 122.7 | unknown |
| W04G3.13 |  | 19.3 | 12.7 | unknown |
| W06D11.2 |  | 22.9 | 24.6 | unknown |
| W07A12.8 |  | 10.3 | 11.8 | unknown |
| Y116F11B.17 |  | 10.2 | 106.5 | unknown |
| Y37H2A.13 |  | 31.1 | 63.4 | unknown |
| Y39B6A.10 |  | 19.5 | 77.5 | unknown |
| Y48E1B.8 |  | 272.9 | 135.2 | unknown |
| Y48G1C.13 |  | 23.9 | 25.8 | unknown |
| Y49F6C.8 |  | 281.0 | 196.7 | unknown |
| Y66C5A.1 |  | 96.6 | 85.2 | unknown |
| Y79H2A.2a |  | 78.4 | 38.2 | unknown |
| Y79H2A.2b.1 |  | 72.9 | 37.8 | unknown |
| ZC266.2 |  | 16.2 | 17.7 | unknown |
| ZK177.1 |  | 45.9 | 169.8 | unknown |
| C18D11.10 |  | 27.5 | 15.2 | unknown |
| K03B8.4 |  | 11.5 | 15.1 | unknown |

^a^ Information about genes was obtained from WormBase (WS229, www.wormbase.org).
